# TGF-β Suppression of HBV RNA through AID-Dependent Recruitment of an RNA Exosome Complex

**DOI:** 10.1371/journal.ppat.1004780

**Published:** 2015-04-02

**Authors:** Guoxin Liang, Guangyan Liu, Kouichi Kitamura, Zhe Wang, Sajeda Chowdhury, Ahasan Md Monjurul, Kousho Wakae, Miki Koura, Miyuki Shimadu, Kazuo Kinoshita, Masamichi Muramatsu

**Affiliations:** 1 Department of Molecular Genetics, Kanazawa University Graduate School of Medical Science, Kanazawa, Japan; 2 Department of Microbiology and Immunology, Columbia University, New York, New York, United States of America; 3 Division of Medical Oncology, Affiliated Zhongshan Hospital of Dalian University, Dalian, China; 4 Evolutionary Medicine, Shiga Medical Center Research Institute, Moriyama, Japan; University of California, San Diego, UNITED STATES

## Abstract

Transforming growth factor (TGF)-β inhibits hepatitis B virus (HBV) replication although the intracellular effectors involved are not determined. Here, we report that reduction of HBV transcripts by TGF-β is dependent on AID expression, which significantly decreases both HBV transcripts and viral DNA, resulting in inhibition of viral replication. Immunoprecipitation reveals that AID physically associates with viral P protein that binds to specific virus RNA sequence called epsilon. AID also binds to an RNA degradation complex (RNA exosome proteins), indicating that AID, RNA exosome, and P protein form an RNP complex. Suppression of HBV transcripts by TGF-β was abrogated by depletion of either AID or RNA exosome components, suggesting that AID and the RNA exosome involve in TGF-β mediated suppression of HBV RNA. Moreover, AID-mediated HBV reduction does not occur when P protein is disrupted or when viral transcription is inhibited. These results suggest that induced expression of AID by TGF-β causes recruitment of the RNA exosome to viral RNP complex and the RNA exosome degrades HBV RNA in a transcription-coupled manner.

## Author Summary

HBV is one of the causative factors of hepatocellular carcinoma. Recent studies have shown that the members of the APOBEC deaminase family are antiviral factors that suppress the replication of viruses, such as HIV-1 and HBV. APOBEC3G suppresses viral replication by either hypermutation of nascent DNA or inhibition of reverse transcription. Recent studies have been suggested that AID, another APOBEC family member, restricts viruses and retrotransposons that use reverse transcription for their replication. However, little is known about the antiviral mechanisms of AID. TGF-β is a pleiotropic cytokine involved in the suppression of HBV replication, but the mechanism underlying its anti-HBV activity is unclear. In this study, we found that AID plays a role in the anti-HBV activity of TGF-β. Further study revealed that AID physically associates with a viral RNP complex containing reverse transcriptase and recruits the RNA degradosome (RNA exosome) to the RNP complex to degrade the viral RNA. To the best of our knowledge, this study is the first to reveal a novel antiviral pathway in which AID triggers viral RNA degradation by tethering the RNA exosome to the viral reverse transcriptase/RNA complex. Viral RNA may be another target for APOBEC antiviral activity.

## Introduction

Hepatitis B virus (HBV) is recognized as the major causative factor of severe liver diseases such as cirrhosis and hepatocellular carcinoma. The clinical outcomes and development of hepatocellular carcinoma and cirrhosis are modulated by viral replication and antiviral immunity against HBV [1]. After entry into the host hepatocyte, HBV forms covalently closed circular DNA (cccDNA) in the nucleus and it initiates the transcription of viral RNAs, including a replicative intermediate known as pregenomic (pg) RNA. Two viral proteins (core and P protein) encapsidate pgRNA to form nucleocapsids, where P protein reverse-transcribes pgRNA to produce relaxed circular (RC)-DNA. These nucleocapsids associate with three types of viral surface proteins for secretion as infectious virions [1,2]. Although the mechanism of HBV replication has been well studied, the mechanisms of antiviral immunity against HBV remain unclear.

Several members of the apolipoprotein B mRNA editing enzyme catalytic polypeptide (APOBEC) family were recently identified as new types of antiviral factors [3–5]. In humans, the APOBEC family comprises at least 11 members, including activation-induced cytidine deaminase (AID), APOBEC 1, 2, 3A, 3B, 3C, 3D, 3F, 3G, 3H, and 4. Most family members deaminate cytidine bases on DNA and/or RNA to generate uridine [3–5]. Accumulating evidence from *in vitro* experiments has further revealed that A3 proteins can inhibit the replication of various types of viruses, including human immunodeficiency virus type 1 (HIV-1) and HBV [4,5]. Among APOBEC deaminases, the molecular mechanism of A3G antiviral activity has been well characterized. In cases of HBV, A3G restricts viral replication through hypermutation and inhibition of reverse-transcription [4,5]. AID is another member of the APOBEC family [4,5] and was originally isolated as a cytidine deaminase that triggered class switch recombination (CSR) and somatic hypermutation (SHM) of transcribed immunoglobulin genes in B cells [6–9]. AID expression was recently shown to be upregulated in human hepatocytes *in vitro* after stimulation with cytokines, including TGF-β1, TNFα, and IL-1β and in the liver in chronic hepatitis patients, and AID involvement in viral infection was suggested [10–17]. Higher serum TGF-β1 levels were reported in some HBV infections *in vivo* [18,19], and TGF-β1 reduces HBV replication *in vitro* [18,20]. However, the precise mechanisms remain elusive. In the present study, we examined the involvement of AID in TGF-β1-mediated restriction of HBV replication. We have demonstrated that TGF-β1 induces AID expression in hepatocytes, which leads to the downregulation of HBV transcripts and inhibition of nucleocapsid formation. AID-dependent downregulation of HBV transcripts requires a viral RNA binding protein (P protein) and RNA exosome components. These data suggest a novel antiviral pathway in which AID recruits the RNA exosome to downregulate viral RNA in HBV infected hepatocytes.

## Results

### TGF-β1-mediated anti-HBV activity

**Fig 1:**
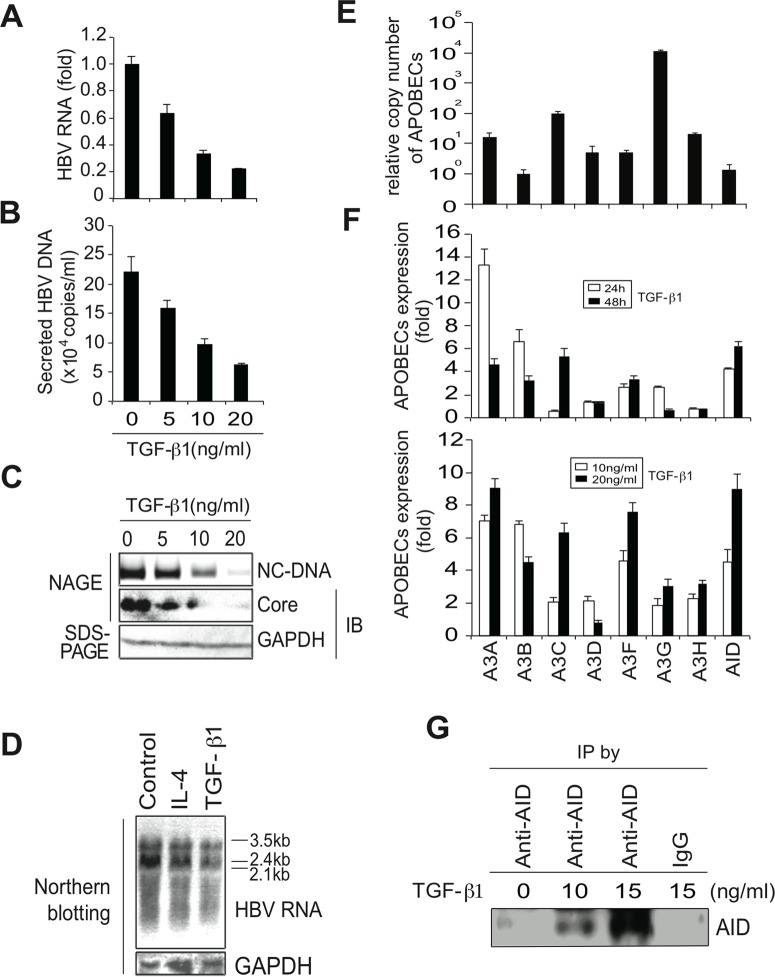
TGF-β1 upregulates APOBEC3 expression and suppresses HBV replication in Huh7 cells. Six hours after transfection of pPB, Huh7 cells were treated with TGF-β1 for 3 days, and HBV replication was evaluated. (A) qRT-PCR shows dose-dependent reduction of HBV transcripts by TGF-β1. (B) NC-DNA levels in secreted virions were also measured using qPCR. (C) Nucleocapsid NC-DNA and core protein levels in crude cytoplasmic extracts were assessed using NAGE assays. GAPDH protein levels in the same crude extracts were determined using western blotting. (D) Huh7 cells were treated with 150 ng/mL IL-4 or 10 ng/ml TGF-β1 for 3 days. Levels of HBV RNA and GAPDH mRNA were determined by Northern blot. Control: non-stimulated Huh7 cells. (E) Relative expression levels of APOBEC deaminases in non-stimulated Huh7 cells; Relative expression levels were determined using qPCR with cDNA from non-stimulated Huh7 cells and standard curves of control APOBEC deaminase DNA. Relative copy numbers of A3B were defined as one. (F) Induction of APOBEC deaminase expression in TGF-β1-treated Huh7 cells was estimated using qRT-PCR. Fold induction of APOBEC deaminases is shown in the top (10 ng/mL TGF-β1 for 24 or 48 h) and bottom (10 or 20 ng/mL TGF-β1 for 24 h) panels. (G) Huh7 cells were treated with indicated concentrations of TGF-β1 for 3 days. AID protein was immunoprecipitated using an anti-AID antibody (or an isotype control IgG, most right) and immunoprecipitated AID protein was determined by western blot. One lane contains immunoprecipitated protein harvested from 60% of 15 cm dish. All data are representative of two to four independent experiments. Error bars represent standard errors of the mean.

To investigate the involvement of APOBEC deaminases in TGF-β1-mediated antiviral activity against HBV, human hepatocytes (Huh7) were transfected with a HBV replicon plasmid (pPB) [21] and the cells were then treated with TGF-β1. Concentrations of 5–20 ng/mL TGF-β1 were used to match the range reported in chronic HBV and hepatocellular carcinoma patients [19]. HBV replication was evaluated by measuring HBV transcript levels using quantitative reverse transcription-polymerase chain reaction (qRT-PCR) (Fig 1A) and Northern blotting (Fig 1D). Viral DNA in secreted virions was determined using qPCR (Fig 1B), and nucleocapsid formation was estimated using native agarose gel electrophoresis (NAGE). Subsequently, cytoplasmic nucleocapsid core protein and nucleocapsid associated DNA (NC-DNA) levels were determined using western blotting and Southern blotting, respectively (Fig 1C). Collectively, TGF-β1 dose-dependently inhibited the production of HBV transcripts, nucleocapsid core protein, and nucleocapsid NC-DNA in both cytoplasmic and secreted samples.

In further experiments, qRT-PCR was used to determine the expression of APOBEC deaminases in the presence and absence of TGF-β1. Initially, relative expression levels of APOBEC deaminases in non-stimulated Huh7 cells were determined. Huh7 cells expressed all APOBEC3 deaminases. A3G and A3C were highly expressed among A3 deaminases (Fig 1E), whereas APOBEC1 expression was not detected in Huh7 cells. In TGF-β1-treated Huh7 cells, expression of most APOBEC deaminases, including A3A, A3B, A3C, A3F, and AID (Fig 1F, upper and lower) was upregulated. Western blotting also detected AID protein in TGF-β1-stimulated Huh7 cells (Fig 1G).

### TGF-β1-mediated reduction of HBV transcripts depends on AID expression

**Fig 2:**
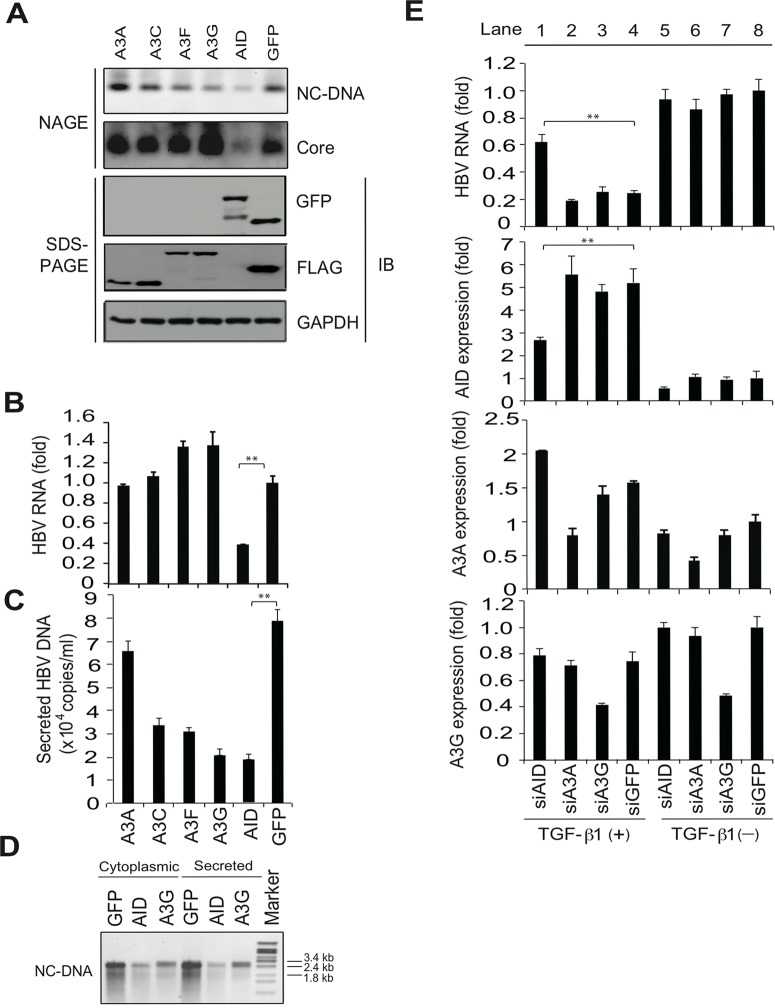
AID is responsible for TGF-β1-mediated reduction of HBV transcripts. To evaluate antiviral activity of indicated APOBEC proteins, Huh7 cells were co-transfected with FLAG-tagged A3A, A3C, A3F, A3G, GFP or GFP-tagged AID expression vectors and pPB. Cells were cultured for 3 days, and then HBV replication was estimated using NAGE assays (A). Protein expression is shown (A, bottom). qRT-PCR analyses of HBV transcripts (B), and qPCR analyses of NC-DNA in secreted virions (C). (D), Secreted virions in the culture medium and cytoplasmic extracts were treated with proteinase K and SDS to digest nucleocapsids, and levels of HBV DNA were determined using Southern blotting. (E), To evaluate contribution of indicated APOBEC proteins, Huh7 cells were co-transfected with pPB and the indicated siRNAs. Six hours later, cells were incubated in the presence or absence of 10 ng/mL TGF-β1. Three days later, total RNA was extracted, and qRT-PCR performed to determine expression levels of HBV transcripts, AID, A3A, and A3G. Although siAID significantly reduced AID expression and prevented the downregulation of HBV transcripts in TGF-β1-stimulated Huh7 cells (lane 1), siA3A and siA3G had no effects against the downregulation of HBV transcripts (lanes 2–4). siGFP was used as a control. Expression levels in lane 8 are defined as one fold induction. **P < 0.01 (*t*-test); Data are representative of two to three independent experiments and error bars represent standard errors of the mean.

It has been demonstrated that APOBEC3 proteins suppress HBV replication *in vitro* [1,4,5]. HBV plasmids and APOBEC deaminase expression vectors were transfected into Huh7 cells, and nucleocaspid formation was estimated using NAGE followed by Southern and western blotting (NAGE assay). The expression of A3G and A3F, but not A3A, reduced NC-DNA levels in cytoplasmic nucleocapsids but did not reduce nucleocapsid core protein levels (Fig 2A). HBV virion DNA was also reduced by A3C, A3G and A3F expression, whereas total HBV transcript levels were not affected by A3C, A3G or A3F (Fig 2B and 2C). It was proposed that minus-strand DNA synthesis was the primary target of A3G-mediated anti-HBV activity in hepatocytes that were transiently transfected with HBV plasmids [1,4,5]. Our results support this proposed mechanism of A3G antiviral activity. In contrast with A3 deaminases, the overexpression of AID reduced HBV transcript levels, nucleocapsid formation, and virion secretion (Fig 2A–2C and S1A and S1B Figs). Nucleocapsid NC-DNA levels were also reduced in AID-expressing cells, as indicated by Southern blotting using purified nucleocapsid NC-DNA (Fig 2D). Importantly, AID expression did not suppress host cell gene transcripts (S2 Fig), suggesting that AID expression may specifically suppress viral RNA. In accordance with the HBV life cycle, these data suggest that AID-mediated reduction of HBV transcripts leads to the downregulation of nucleocapsid core protein and NC-DNA.

To investigate the contributions of APOBEC deaminases to TGF-β1-mediated anti-HBV activity, small interfering (si) RNAs targeting specific deaminases were transfected with the HBV plasmid into Huh7 cells. Cells were further treated with TGF-β1 to assess the effects on TGF-β1-mediated reduction of HBV transcripts. TGF-β1 stimulation in siGFP-transfected control cells reduced HBV transcript levels by 76% compared with non-stimulated cells (Fig 2E, top, lane 4 vs. 8). Transfection of siAID, siA3A, or siA3G suppressed the corresponding endogenous genes by up to 51%, 40%, and 56%, respectively. However, the knockdown of A3A and A3G did not affect TGF-β1-mediated reduction of HBV RNA in comparison with the siGFP control. In contrast, TGF-β1-mediated downregulation of HBV RNA was significantly attenuated by the knockdown of AID (Fig 2E, top, lane 1 vs. 4). These data suggest that TGF-β1-mediated downregulation of HBV transcripts is dependent on endogenous AID expression. Partial rescue of HBV transcript levels in siAID-transfected cells also suggests the involvement of either residual AID or other unidentified effectors in TGF-β1-mediated reduction of HBV transcripts.

### AID expression levels required for initiating class switching are sufficient for AID-mediated reduction of HBV transcripts

**Fig 3:**
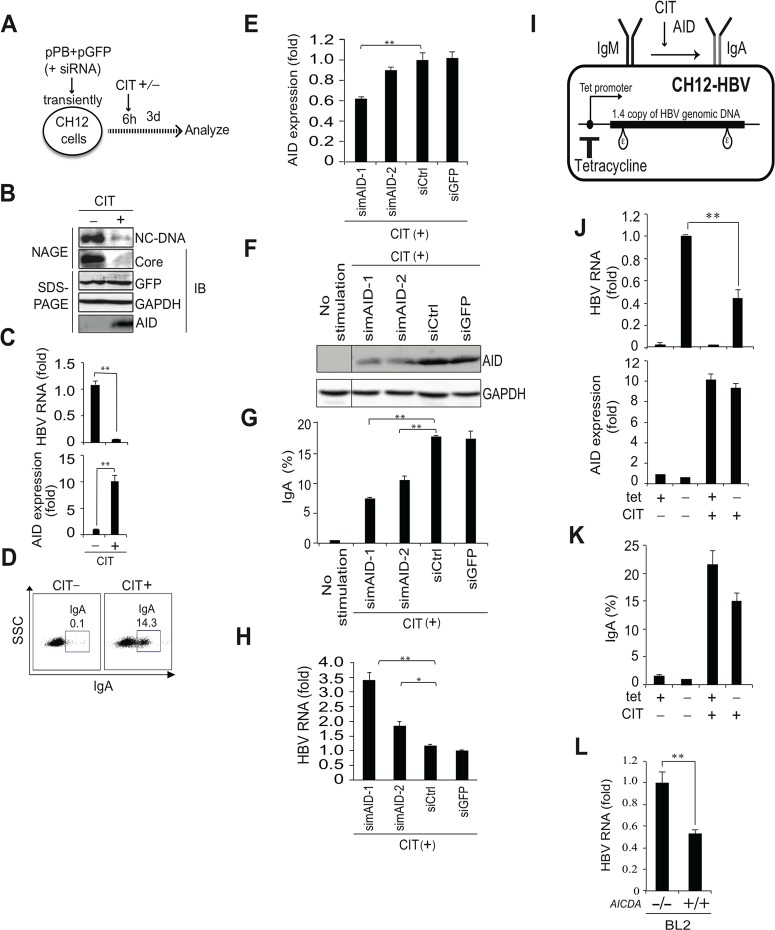
IgA switching activity correlates with reduction of HBV transcripts in B cells. (A, B, C, D) pPB and GFP expression vectors were transiently co-transfected into a mouse B cell line (CH12F3-2). Six hours after transfection, cells were divided into two groups and stimulated with (or without) CD40 ligand, IL-4, and TGF-β1 (CIT) for 3 days to induce IgA switching; (A) Schematic diagram of experimental design; (B) Nucleocapsid formation was measured using NAGE assays and GFP expression was used to confirm transfection. (C) HBV transcripts and AID expression levels were determined using qRT-PCR. (D) AID dependent IgA switching was determined using flow cytometry. (E, F, G, H) CH12F3-2 cells were co-transfected with pPB and the indicated siRNA against mouse AID (simAID-1 and -2) or controls (siCtrl and siGFP), and after 6 hours incubation, cells were further stimulated with CIT for 3 days. HBV transcript levels, knock down efficiency of AID, and IgA switching were determined using qRT-PCR, western blotting, and flow cytometry, respectively. (I, J, K) A tetracycline promoter-regulating HBV plasmid (pTre-HBV) was stably transfected into CH12F3-2 transfectants expressing tetracycline-responsible transactivator (Tet-off). Established CH12F3-2 transfectants were designated CH12-HBV; (I) Schematic diagram of CH12-HBV; (J) CH12-HBV cells were incubated in the presence or absence of CD40 ligand, IL-4, or TGF-β1 (CIT) and tetracycline as indicated for 2 days to induce endogenous AID expression and IgA switching. HBV transcription and AID expression levels were determined using qRT-PCR. (K) IgA switching was detected according to surface expression of IgA using flow cytometry. (L) AICDA-deficient and-wild type BL2 cells were transfected with HBV plasmid (pPB), and qRT-PCR was performed at 3 day post-transfection. *P < 0.05, **P < 0.01 (*t*-test). Data are representative of two to three independent experiments and error bars represent standard errors of the mean.

We previously demonstrated that the induction of AID in B cells triggers class switch recombination (CSR) in immunoglobulin genes [7–9], which validates B cells as a model to study AID functions. In addition, it is anticipated that peripheral blood mononuclear cells and B cells can be extrahepatic reservoirs for HBV infection [22,23]. Thus, we investigated whether endogenous AID expression that could trigger CSR is also sufficient to trigger a reduction in HBV transcripts. AID expression and IgA class switching can be induced in CH12F3-2 mouse B cells following co-stimulation with CD40 ligand, IL-4, and TGF-β1 (designated CIT) [6,24]. CH12F3-2 cells transiently transfected with the HBV plasmid were divided into two groups, and were treated with (or without) CIT to induce IgA switching, a GFP expression vector was co-transfected to verify transfection efficiency. At three days post-transfection, HBV replication and CSR were determined (Fig 3A–3D), and showed that CIT induced AID protein expression and initiated IgA class switching, as previously reported [6,24]. Moreover, NAGE assays and qRT-PCR revealed that HBV transcripts, nucleocapsid NC-DNA, and core protein were downregulated in CIT-stimulated cells, whereas the expression of GFP remained intact after CIT stimulation (Fig 3B and 3C). These data indicate that CIT stimulation specifically inhibits HBV replication in mouse B cells. We further used siRNAs against mouse AID (simAID-1 and -2) to assess the contribution of AID to the suppression of HBV products in CIT-stimulated cells. Although simAIDs knocked down endogenous AID transcripts to only 39% determined by qRT-PCR (Fig 3E), western blotting revealed clear suppression of endogenous AID protein levels (Fig 3F). Furthermore, flow cytometric analyses revealed that IgA class switching is attenuated by the knockdown of AID (Fig 3G), and qRT-PCR revealed that HBV transcript levels are inversely correlated with AID expression and IgA switching efficiency (Fig 3G and 3H). To avoid artifacts due to the transfection process, a tetracycline-dependent stable line of the HBV replicon plasmid was established in CH12F3-2 cells (CH12-HBV; Fig 3I). CH12-HBV cells were treated with CIT to induce IgA switching, and HBV transcript levels were determined. Subsequent qRT-PCR analyses demonstrated significant reductions of HBV transcript levels upon IgA switching (Fig 3J and 3K). These data clearly demonstrate that endogenous AID expression sufficient to trigger CSR is also sufficient to downregulate HBV transcripts.

Another putative activity of AID involves the initiation of somatic hypermutation (SHM) in immunoglobulin variable genes [8,9,25] previously demonstrated that human BL2 B cells autonomously induce SHM, which is absent following AID gene disruption by gene targeting. Thus, we transiently transfected the HBV replicon plasmid into BL2 cells and compared HBV replication in *Aicda*+/+ and *Aicda*−/− BL2 cells. We previously demonstrated that nucleocapsid NC-DNA and core protein are suppressed in *Aicda*+/+ in comparison with *Aicda*−/− BL2 cells, although co-transfected GFP expression levels were similar in both cell types [26]. Using identical samples, we here showed that HBV transcript levels in *Aicda*+/+ BL2 cells were almost 50% of those in *Aicda*−/− BL2 cells (Fig 3L).

Both mouse and human B cell lines collectively demonstrated that endogenous AID activity that can initiate either CSR or SHM of immunoglobulin genes is sufficient to trigger downregulation of HBV transcripts.

### AID-mediated downregulation of HBV transcripts requires intact P protein structure

**Fig 4:**
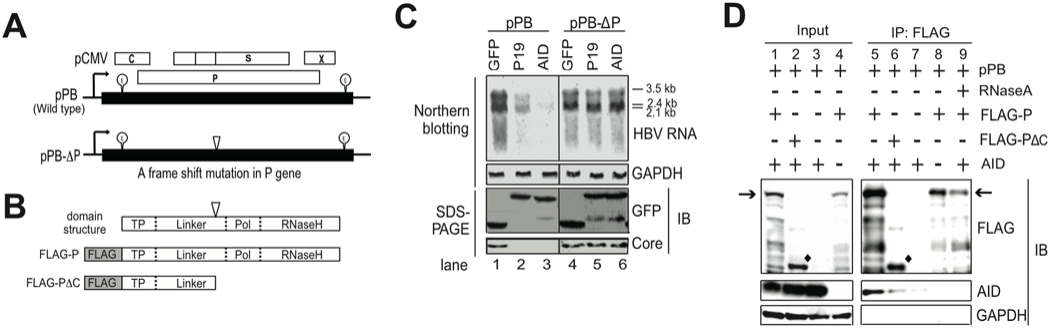
Intact P protein is required for AID-mediated downregulation of HBV transcripts and AID associates with HBV P protein. (A) Schematic diagram of wild-type and mutant HBV replicon plasmids. Partially redundant HBV genomic DNA is shown as black boxes and the positions of 5′-ε and 3′-ε are shown. Open reading frames corresponding to C, P, S, and X genes are shown as open boxes. The position of the frame-shift mutation in the mutant replicon plasmid (pPB-ΔP) is indicated as an open triangle. This frame-shift mutation results in loss of the C-terminal portion (polymerase and RNase H domains) from the P protein; pCMV, CMV promoter. (B) Schematic diagram of P protein domain structure; (C) Replicon plasmid (pPB or pPB-ΔP) and GFP fusion expression vectors (mock, AID, and p19-mutant AID) were transfected into Huh7 cells, and after four days, AID-mediated downregulation of HBV transcripts was compared between two replicon plasmids or between wild-type and p19 mutant AID using northern blotting. Expression of HBV core and GFP fusion proteins (mock, AID, and p19-mutant AID) was confirmed using SDS-PAGE and western blotting. (D) Wild-type replicon plasmid (pPB) and indicated protein expression vectors (FLAG-P, FLAG-PΔC, or AID) were transfected into Huh7 cells. Three days later, physical associations between AID and FLAG-P (or FLAG-PΔC) proteins were determined using immunoprecipitation (IP). In lane 9, crude extract was incubated with RNase A before immunoprecipitation. Positions of FLAG-P and FLAG-PΔC proteins are indicated by arrows and diamonds, respectively. The structure of FLAG-PΔC protein is shown in B. Input; crude extract. Data are representative of two to three independent experiments.

To investigate the mechanism of AID-mediated downregulation of HBV transcripts, we initially focus on the viral P protein, because AID, P protein and HBV transcripts form RNP complex [26]. In these experiments, we applied a mutant HBV replicon plasmid (pPB-ΔP, Fig 4A) that expresses a mutant P protein lacking the C-terminal half including catalytic DNA polymerase and RNase H domains [26]. Transfection with pPB-ΔP did not support nucleocapsid DNA synthesis due to inhibition of reverse-transcription, although HBV transcription and core protein synthesis remained intact in Huh7 cells (Fig 4C, lanes 1 and 4). AID-mediated downregulation of HBV transcripts was compared between pPB- and pPB-ΔP-transfected Huh7 cells. As shown in Fig 4C, AID-mediated downregulation of HBV transcripts was not observed in pPB-ΔP-transfected Huh7 cells, indicating that AID-mediated downregulation of HBV transcripts requires intact viral P protein.

The requirement of cytidine deaminase activity for AID was also investigated. AID mutant P19 was isolated from a class switch deficient patient and the deaminase activity was negligible owing to a missense mutation in catalytic cytidine deaminase domain [27]. P19 was then co-transfected with the wild-type HBV plasmid, and HBV transcript levels were compared with that in wild-type AID controls. These experiments showed that the P19 mutant significantly reduced HBV transcript level, although less effectively than wild-type AID (Fig 4C). Therefore, under experimental conditions of AID over-expression, cytidine deaminase activity is not exclusively required for AID-mediated downregulation of HBV transcripts.

In subsequent experiments, we generated an expression vector (pFLAG-PΔC) for the mutant P protein which was a corresponding mutant P protein produced from pPB-ΔP-transfected cells (Fig 4B). Then the physical association between AID and the mutant P protein was examined. Immunoprecipitation analyses showed that wild type P protein co-precipitated AID in an RNase A-sensitive manner (Fig 4D, lane 5, 8, 9), whereas the mutant P protein (FLAG-PΔC) precipitated only trace levels of AID protein, suggesting that AID may not efficiently form RNP complex with the mutant P protein in pPB-ΔP-transfected cells. To explore which subcellular sites are responsible for AID and P protein interaction, cells were biochemically fractionated into three fractions (cytoplasmic, soluble nuclear, and insoluble nuclear) (S3 Fig). Immunoprecipitation analyses using cytoplasmic and soluble nuclear proteins revealed that AID can associate with P protein in both nucleus and cytoplasm. It is of note that robust signals of AID and P proteins were found in the insoluble fraction that contains chromatin and other nuclear proteins.

### AID-mediated downregulation of HBV transcripts requires the RNA exosome complex

**Fig 5:**
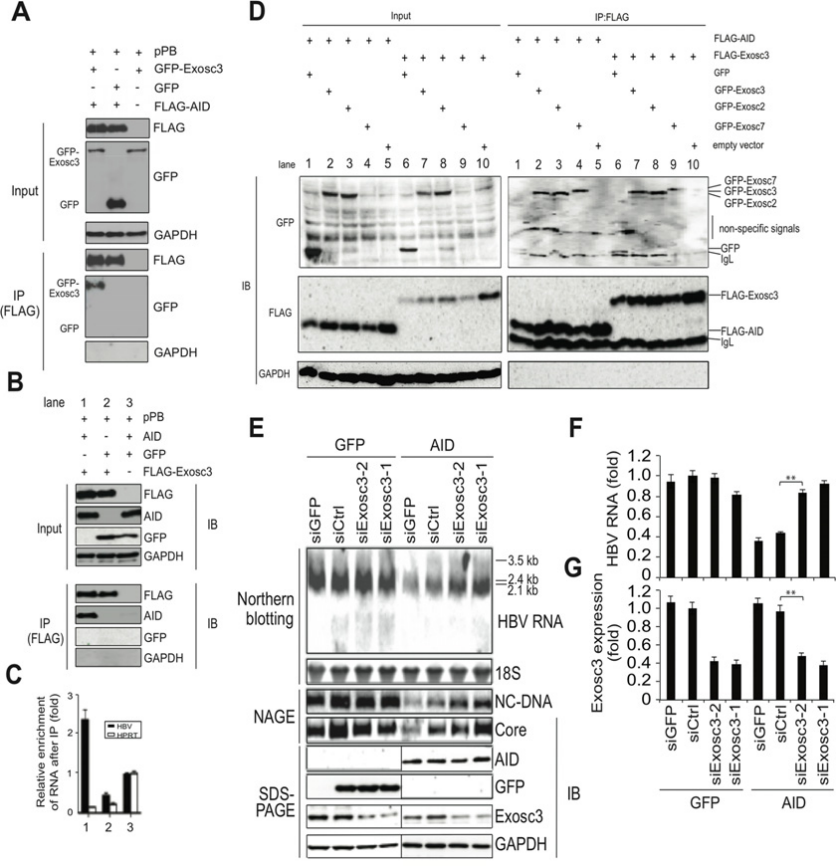
AID inducing HBV RNA reduction depends on Exosc3. (A, B) Huh7 cells were co-transfected with pPB and the indicated protein expression vectors, and were cultured for 3 days. Crude extracts (input) were then subjected to IP using an anti-FLAG antibody, and crude extracts and IP fractions were analyzed using western blotting. (C) Fold enrichment of HBV or HPRT transcripts upon anti-FLAG-Exosc3 IP; To determine RNA coprecipitation with the RNA exosome component Exosc3, Huh7 cells were transfected with pPB, pFLAG-Exosc3, and pCMV-AID (or pEGFP-C2), and were cultivated for 3 days. IP using anti-FLAG antibody was then performed, complexes of FLAG-Exosc3 were then eluted using free FLAG peptides, and the eluted RNA was analyzed using qRT-PCR. Combination of expression vectors used for transfection is the same with B (see numbers below the graph), and values in lane 3 were defined as 1. Error bars represent standard errors of the mean. (D) Associations of AID with RNA exosome proteins; Huh7 cells were co-transfected with indicated expression vectors, and were cultured for 3 days. Crude extracts (input) were subjected to IP with FLAG antibody, and crude extracts and IP fractions were analyzed using western blotting. Expression levels of GFP-Exosc7 were too low to be visualized in the crude extract (lanes 4 and 9, input), but GFP-Exosc7 was clearly detectable after FLAG-AID and FLAG-Exosc3 immunoprecipitation (lanes 4 and 9, IP). (E) Huh7 cells were co-transfected with pPB and either AID or GFP expression vectors and each of the siRNAs indicated in E and F, and cells were cultured for 3 days. HBV transcript levels, nucleocapsid formation, and Exosc3 expression were estimated using northern and western blotting, NAGE assays (E), and qRT-PCR analyses (F and G); siGFP and siCtrl were used as controls; **P < 0.01 (*t*-test); Data are representative of two to three independent experiments and error bars represent standard errors of the mean.

AID was recently shown to physically interact with RNA exosome proteins and promote CSR in transcribed immunoglobulin genes [28,29]. The RNA exosome comprises a ring-like structure and two catalytic components, and plays a major role in various RNA processing and degradation pathways [30,31]. Exosome component 3 (Exosc3, also known as Rrp40) is non-catalytic but is essential for the degradation and processing of target RNA, and the knockdown of Exosc3 severely diminished the RNA exosome function [32]. Thus, we investigated whether Exosc3 is involved in TGF-β1-mediated downregulation of HBV transcripts in Huh7 cells. As shown in Fig 5A, immunoprecipitation of AID co-purified Exosc3, but did not precipitate GAPDH or GFP. Exosc3 immunoprecipitation also co-purified AID but not GAPDH or GFP (Fig 5B), indicating a physical association between AID and Exosc3 proteins. This study found a physical association between AID and the RNA exosome proteins (Exosc 2, 3, 7) in Huh7 cells in the absence of HBV replication (Fig 5D). As expected, Exosc3 immunoprecipition also copurified with other RNA exosome proteins (Exosc2 and 7) in Huh7 cells (Fig 5D). Furthermore, we found that AID can also associate with RNA exosome in both nucleus and cytoplasm (S4A Fig). Consistent with AID-RNA exosome interaction, RNA exosome proteins localized to both cytoplasm and nucleus (S5A and S5B Fig). We previously demonstrated a physical association between HBV transcripts and AID in HBV-replicating Huh7 cells [26]. In current study, we examined whether Exosc3 associates with HBV transcripts. As shown in Fig 5C, qRT-PCR analysis demonstrated enrichment of HBV but not HPRT transcripts in Exosc3 immunoprecipitates, which was observed only when AID was present (Fig 5C, lane 1). This is also true when nuclear or cytoplasmic Exosc3 was separately precipitated (S4B Fig). AID-mediated downregulation of HBV transcripts was observed in both nucleus and cytoplasm, and efficiency of downregulation was comparable between nucleus, cytoplasm, and whole cell samples (S6A and S6B Fig). These results suggest that AID recruits the RNA exosome proteins to HBV transcripts and AID downregulates HBV RNA in nucleus.

**Fig 6:**
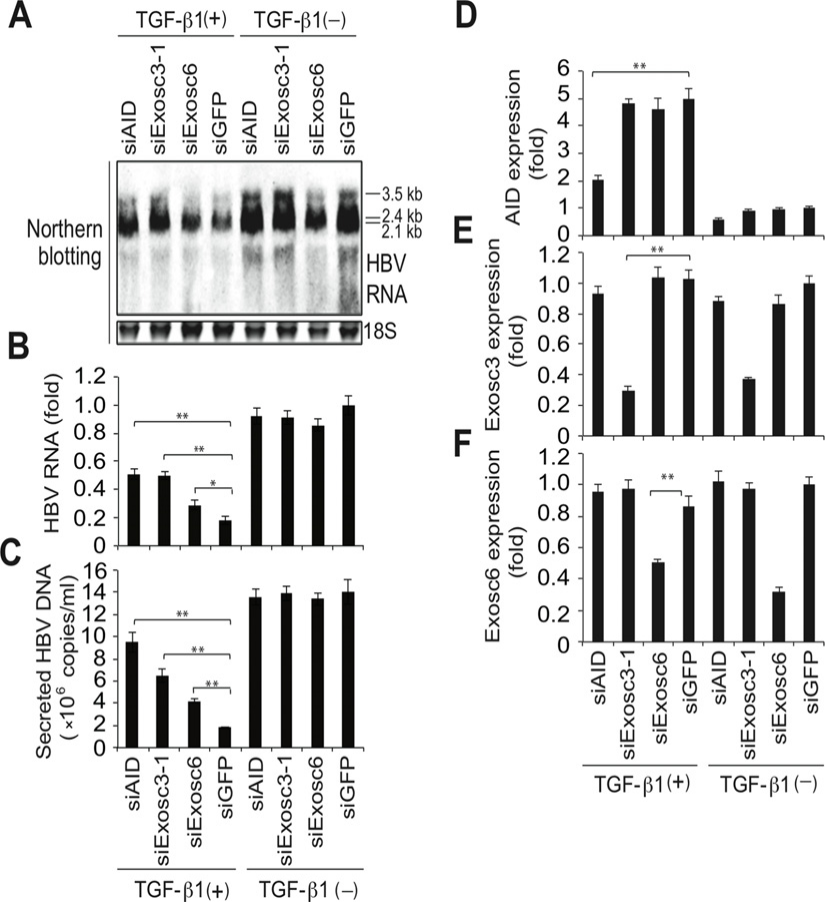
TGF-β1-mediated downregulation of HBV transcripts requires RNA exosome proteins. Huh7 cells were transfected with pPB and indicated siRNAs. Six hours after transfection, the cells were incubated in the presence or absence of 10-ng/mL TGF-β1 for 3 days. Total RNA was analyzed using northern blotting (A) and qRT-PCR to determine HBV transcription of AID, Exosc3, and Exosc6 (B, D, E, F). In C, NC-DNA from secreted virions was also measured by qPCR. Transfection of siAID and siExosc3 partially restored TGF-β1-mediated downregulation of HBV transcripts and viral production; *P < 0.05, **P < 0.01 (*t*-test); error bars represent standard errors of the mean. Data are representative of two to three independent experiments.

**Fig 7:**
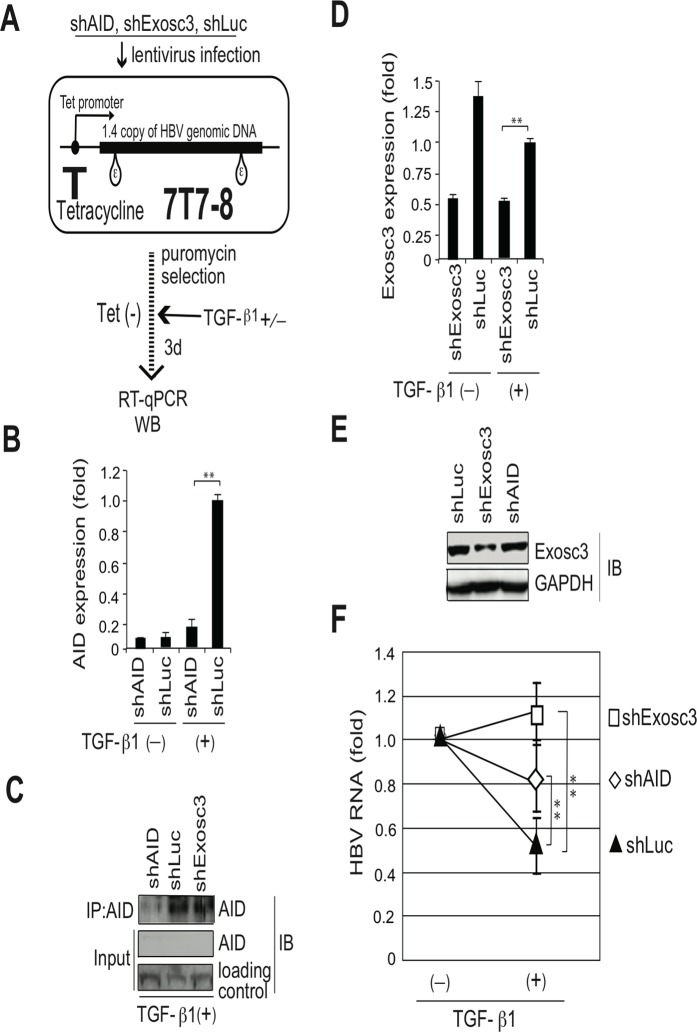
TGF-β1-mediated reduction of HBV transcripts depends on AID and Exosc3. Stable HBV transfectant Huh7 cells (7T7-8) were infected with recombinant lentiviruses to express indicated short hairpin (sh) RNA, and then cells were incubated in the presence or absence of 15 ng/ml TGF-β1 for 3 days. (A) Schematic diagram of experimental design; (B) AID expression levels in qRT-PCR and (C) IP western blotting. Crude extract before IP was also blotted (input). Crude extracts from TGF-β1-treated 7T7-8 transfectants were immunoprecipitated by anti-AID antibody. Loading control: anti-(adenosine deaminase acting on RNA) ADAR. (D) Exosc3 expression level in qRT-PCR or western blotting (E); shLuc was used as a control; (F) Reductions of HBV transcript levels following TGF-β1 treatment are compared between shAID-, shExosc3-, and shLuc-expressing 7T7-8 cells. HBV transcript levels of each non-stimulated transfectant are defined as 1; shLuc was used as a non-targeted control. *P < 0.05, **P < 0.01 (*t*-test), error bars represent standard errors of the mean. Data are representative of two to three independent experiments.

To further confirm that the RNA exosome is involved in AID-mediated downregulation of HBV transcripts, we used the siRNA knockdown of Exosc3, which is essential for the RNA exosome function [32]. In these experiments, siRNAs against Exosc3 were co-transfected with the HBV plasmid and AID (or GFP) expression vectors, and HBV replication was determined. Northern blotting, NAGE assays, and qRT-PCR analyses showed the attenuation of AID-mediated downregulation of HBV transcripts and nucleocapsid formation in siExosc3 transfectants (Fig 5E–5G). In contrast, AID, GFP, and GAPDH expression were not affected by Exosc3 depletion (Fig 5E, bottom). Importantly, knock down of Exosc3 did not increase HBV RNA levels in GFP transfected samples. Moreover, siExosc3 transfection attenuated TGF-β1-mediated downregulation of HBV transcripts and nucleocapsid formation in a similar manner to that observed after transfection with siAID (Fig 6A–6F). In further experiments, knockdown of another RNA exosome component Exosc6 also attenuated TGF-β1-mediated downregulation of HBV transcripts and nucleocapsid formation, albeit less effectively than the knockdown of siExosc3 and AID (Fig 6A–6F). Similarly, the contributions of AID and Exosc3 to TGF-β1-mediated downregulation of HBV transcripts were examined in stably HBV-transfected Huh7 cells (7T7-8) [26]. The short hairpin (sh) RNA expressing lentivirus was transduced into 7T7-8 cells, and two stable transfectants (shAID and shExosc3) and a control transfectant (shLuc) were established after puromycin selection. These cells were then cultured in the presence or absence of TGF-β1 (Fig 7A). Subsequent qRT-PCR and western blotting showed reduced endogenous AID and Exosc3 expression (Fig 7B–7E). Comparison of HBV transcript levels between TGF-β1-treated and non-treated 7T7-8 cells revealed that TGF-β1-mediated reduction of HBV transcripts is restored by the knockdown of AID and Exosc3 (Fig 7F). Taken together, these data indicate that RNA exosome proteins (Exosc3 and Exosc6) and AID are required for TGF-β1-mediated downregulutation of HBV transcripts.

### AID-mediated downregulation of HBV transcripts depends on transcription

**Fig 8:**
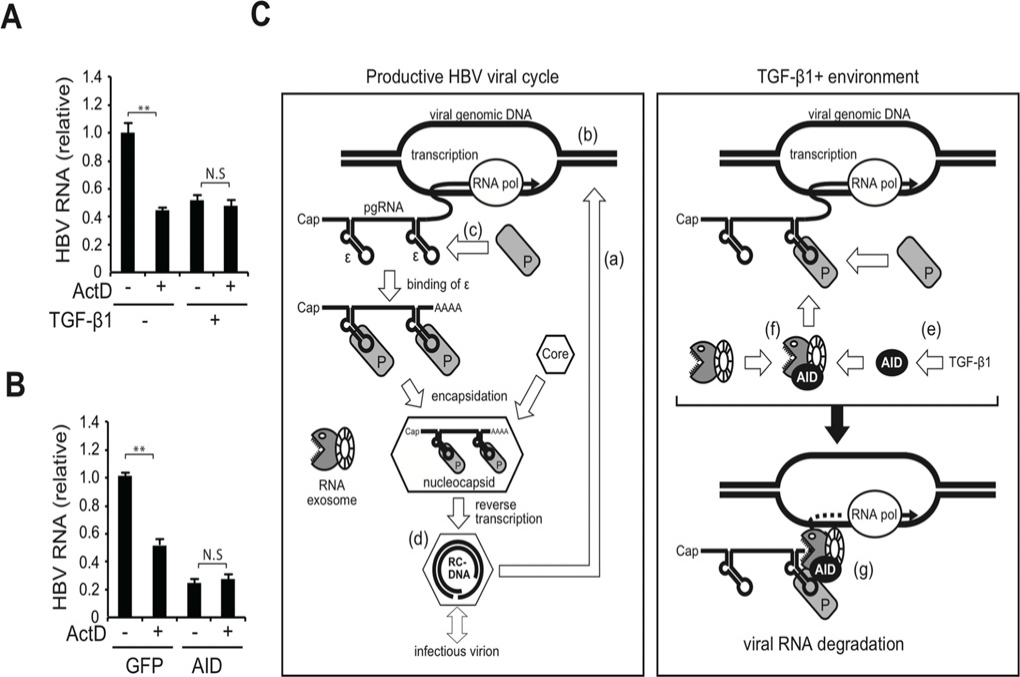
Transcription dependency for TGF-β1-mediated reduction of HBV transcripts and a proposed model. HBV-expressing 7T7-8 cells were treated with 10 ng/ml TGF-β1 (A) or transfected with AID (or GFP) expression plasmid (B) and cultivated for 3 days. At 18 h before harvest, 100 ng/ml actinomycin D (ActD) was added to block transcription. Total RNA was extracted to measure HBV RNA levels (normalized by HPRT) by qRT-PCR. HBV RNA levels in non-treated (A) and GFP transfected cells (B) were taken as one. **P < 0.01 (*t*-test); Data are representative of two independent experiments and error bars represent standard errors of the mean. (C) Hypothetical model: Left panel, the canonical HBV life cycle; (a) After the entry of HBV into a hepatocyte, nucleocapsid NC-DNA is converted into cccDNA. (b) Subsequently, cccDNA expresses viral transcripts, including pgRNA, pre-S1, pre-S2/S, X, and pre-C mRNAs. In this study, most viral RNAs were transcribed from the HBV plasmid instead of cccDNA. All transcripts possess the 3′-ε RNA stem-loop structure; only pgRNA is shown. (c) P protein binds to the ε structure and stabilizes it, and the core protein (indicated by hexagons) is then recruited to form the nucleocapsid. (d) Inside the nucleocapsid, P protein reverse transcribes pgRNA to produce NC-DNA. A mature nucleocapsid gains S proteins and is secreted as an infectious virion. The minor fraction of nucleocapsid may enter a second intracellular viral cycle. (e) Right panel, TGF-β1 stimulation of hepatocytes induces AID expression. (f) AID associates with the RNA exosome proteins. The RNA exosome comprises ring-like core and exonuclease catalytic components. AID associates with HBV transcripts and P proteins. (g) Consequently, AID bridges the RNA exosome with the RNP complex of HBV transcripts and P protein, which may trigger the degradation of HBV transcripts.

Immunoglobulin gene diversification triggered by AID is coupled with the transcription of immunoglobulin locus [8,9]. Here we examined whether AID-mediated HBV RNA downregulation is also coupled with transcription using a transcription inhibitor actinomycin D (ActD). Using a stable HBV transfectant (7T7-8), we generated experimental conditions in which endogenous or ectopic AID is expressed in HBV-replicating cells. ActD was then added to evaluate whether it could downregulate HBV RNA even in ActD-treated cells. As shown in Fig 8A and 8B, no significant synergistic reduction in HBV RNA levels by ActD and AID was observed in TGF-β1-treated and AID-overexpressing cells, indicating that AID was unable to reduce HBV RNA levels in ActD-treated cells. These results suggest that AID-mediated HBV RNA downregulation depends on transcription, similar to the immunoglobulin gene diversification triggered by AID.

## Discussion

AID is a key molecule involved in the diversification of immunoglobulin genes [8,9], and thus its role in B cells is well understood. AID expression has been also found in non-B cells [11–13], however, its role in non-B cells remains elusive. In the present study, we assessed AID involvement in TGF-β1-dependent anti-HBV activity and demonstrated the following: (1) AID expression is upregulated in TGF-β1-stimulated hepatocytes and reduces HBV RNA levels (Figs 1 and 2); (2) TGF-β1-mediated downregulation of HBV transcripts is inhibited by AID knockdown (Fig 2); and (3) endogenous AID protein levels in B cells capable of inducing immunoglobulin diversification also downregulate HBV transcript levels in a transcription-coupled manner (Figs 3 and 8). These data indicate that AID is involved in a TGF-β1-mediated anti-HBV pathway.

Which part of the virus life cycle that is targeted by AID-mediated downregulation of HBV transcripts? Another APOBEC protein, A3A, which was previously proposed to hypermutate transfected plasmids in human peripheral monocytes [33]. However, AID did not change HBV transcript levels in hepatocytes transfected with the mutant HBV replicon (pPB-ΔP) (Fig 4C). In contrast, HBV transcripts in hepatocytes transfected with the wild-type replicon (pPB) were specifically downregulated by following the expression of AID expression (Figs 2 and 4). Intact HBV transcript levels in AID-expressing pPB-ΔP transfectants suggest that AID-mediated reduction of HBV transcripts is not due to plasmid targeting or promoter interference by AID activity. Otherwise, targeting of HBV plasmid or promoter activity would result in reduction of HBV transcripts in both pPB- and pPB-ΔP-transfectants because those HBV plasmids share the exactly same DNA sequences except 4 base insertion within *P* gene in pPB-ΔP. Previous our study demonstrated that chicken AID can downregulate cccDNA of duck hepatitis virus in a uracil-DNA glycosylase (UNG)-dependent manner [34], therefore, the next obvious candidate for AID target is cccDNA of HBV. We determined cccDNA levels of transfectants using the rolling circle amplification (RCA) assay, which specifically amplifies circular DNA, including cccDNA. As per our results, cccDNA was clearly detected in a cccDNA-producing control cell line (HepG2.2.5) [10–15,35]; however, the HBV-replicating transfectants used in this study rarely produced cccDNA (S7A and S7B Fig). Therefore, the majority of the HBV transcripts produced from HBV transfectants in the present experimental systems are derived from HBV replicon plasmids and not from cccDNA. That means that targeting of cccDNA does not explain the observed downregulation of HBV transcripts in the present experimental systems. AID over-expression was previously shown to deaminate nucleocapsid NC-DNA and encapsidated pgRNA [10,13,26]. However, because NC-DNA is reverse transcribed from HBV pgRNA, AID activity against NC-DNA fails to explain the downregulation of HBV transcripts. Reduction of HBV RNA by the catalytically dead mutant AID (p19) indicates that encapsidated pgRNA editing is distinct from AID-mediated reduction of HBV RNA. Thus, we concluded that AID directly targets HBV transcripts.

The viral P protein is a reverse transcriptase that binds 5′-ε RNA structure in pgRNA and encapsidates pgRNA to the nucleocapsid [1,2] (see also S1 Fig). It is demonstrated that P protein can also bind 3′-ε RNA structure present in 2.4-, 2.1-, and 0.7-kb viral mRNAs [36], indicating that P protein binds all types of HBV transcripts. AID and TGF-β downregulate HBV transcripts containing 3′-ε but not cellular transcripts (Figs 1D, 4C, 5E and 6A, and S1 and S2 Figs). AID-mediated HBV RNA reduction did not occur in hepatocytes expressing a mutant P protein (Fig 4C). We demonstrated that AID can physically associate with viral RNP complexes comprising P protein [26] (Fig 4). Therefore, AID-mediated HBV RNA reduction is dependent on the presence of intact P protein and P protein may determine the target specificity for AID-mediated HBV RNA reduction.

Mao *et al*. recently reported that zinc finger antiviral protein (ZAP) inhibits the replication of HBV by binding the 5′-ε RNA structure of HBV and degrading viral RNA [37]. To explore the molecular mechanism of AID-mediated downregulation of HBV transcripts, we first investigated the possible involvement of ZAP. RT-qPCR revealed that AID expression did not affect the level of ZAP mRNA (S2 Fig). Knocking down of ZAP by transfection of siRNAs against ZAP increased HBV RNA levels, which indicates that ZAP reduces the basal level of HBV RNA; however, AID-mediated downregulation of HBV transcripts was not affected by knocking down of ZAP expression (S8 Fig). These results imply that the ZAP antiviral pathway is dispensable for AID-mediated downregulation of HBV transcripts.

Next, we explored the possible involvement of the RNA exosome. Basu *et al*. [29] demonstrated that AID binds and recruits the RNA exosome complex to R-loop structures in immunoglobulin genes. Here, we investigated whether AID forms a complex with RNA exosome proteins in hepatocytes. The immunoprecipitation of AID and Exosc3 revealed the formation of a RNP complex comprising AID and RNA exosome proteins in both nucleus and cytoplasm of hepatocytes, and that HBV transcripts formed a specific complex with the RNA exosome in an AID-dependent manner (Fig 5 and S4 Fig). Furthermore, AID-dependent downregulation of HBV transcripts was inhibited in the absence of the essential RNA exosome component Exosc3 (Fig 5). We also demonstrated that AID-mediated downregulation of HBV transcripts does not occur when P protein loses the C-terminus domain, which is essential for AID binding (Fig 4C). Inhibition of transcription resulted in blocking of AID-mediated downregulation of HBV transcripts (Fig 8). Taken together, we suggest that AID recruits the RNA exosome to transcribing HBV RNA through an association with the P protein, and thereby downregulates HBV transcripts (Fig 8C).

AID has been shown to reduce the transpositioning of the reverse transcriptase-dependent retroelement L1 [14,15]. Moreover, MacDuff et al. demonstrated that a catalytically dead mutant and wild-type AID suppress L1 transpositioning. Here, we showed that the AID-mediated HBV RNA reduction depends on HBV reverse transcriptase (P protein), and catalytically dead mutant AID (p19) reduces HBV transcript levels (Fig 4). It would be interesting to examine whether suppression of transpositioning by AID is also dependent on the RNA exosome.

To our knowledge, this is the first study to show that AID mediates the downregulation of viral RNA through the RNA exosome complex. However, further studies are required to elucidate the mechanisms of AID-mediated HBV RNA downregulation, and to investigate the involvement of AID in anti-HBV activity *in vivo*.

## Materials and Methods

### NAGE assays

NAGE assays were performed as previously described [20,26,38,39]. In brief, intact nucleocapsid particles were separated from crude extracts of HBV-replicating cells using agarose gel electrophoresis. Nucleocapsid particles within the gel were then denatured under alkaline conditions, and were transferred onto nitrocellulose membranes (Roche). Nucleocapsid DNA and core proteins were detected using Southern and western blotting with a double-stranded HBV DNA probe spanning the whole viral genome and an anti-core antibody, respectively.

### Cell culture and transfection

Plasmids were transfected into Huh7 cells using CalPhos (Clontech) or Fugene 6 (Roche). The total transfected plasmid per sample was normalized by supplementation with empty or GFP expression plasmids. Co-transfection of plasmid and siRNA was performed using lipofectamine 2000 according to the manufacturer’s instructions. Stealth-grade siRNA for mouse and human AID, A3A, A3G, Exosc3, Exosc6, and control were purchased from Invitrogen. In all transfection experiments, control siRNA was designed to differ from all mammal transcripts. BL2 [25] and CH12F3-2 cell culture, CIT stimulation, and transfection by electroporation were performed as previously described [24–26,40]. The HBV-replicating Huh7 cell line (7T7-8) was established and described previously [26]. The pTre-HBV [41] vector was transfected into tetracycline activator expressing CH12F3-2 cells (FTZ14 [42]) to establish the CH12-HBV cell line. Subsequently, shLuc, shAID, and shExosc3 expressing 7T7-8 cells were established by infection with recombinant lentivirus followed by puromycin selection. Recombinant lentiviruses were generated by transient transfection of shLuc-, shAID-, and shExosc3-pLKO1-puro and packaging plasmids (pMD2.D and psPAX2, Addgene plasmid 12259 and 12260, respectively, kind gifts of Dr. Trono) in 293T cells according to the manufacturer’s instructions.

### Expression vectors and reagents

Human TGF-β1 and IL-4 were purchased from R&D systems. Actinomycin D was purchased from Sigma-Aldrich. The HBV replicon plasmid (pPB) contains 1.04 copies of HBV genomic DNA and expresses pgRNA under the control of the CMV promoter [21]. The pPB-ΔP plasmid contains a frame-shift mutation in codon 306 of the *P* gene, leading to loss of the C-terminal 539 amino acids, which comprise catalytic and RNase H domains [26]. Probe labeling and northern and Southern blots were developed using the AlkPhos direct labeling system (Amersham). Signals for northern, Southern, and western blots were analyzed using a LAS1000 Imager System (FujiFilm). Other expression vectors are listed in S1 Table and the gene accession numbers were listed in S3 Table.

### Immunoprecipitation and western blotting

Cells were lysed in buffer containing 50-mM Tris-HCl (pH 7.1), 20-mM NaCl, 1% NP-40, 1-mM EDTA, 2% glycerol, and protease inhibitor cocktail (Roche). After centrifugation, supernatants were incubated with the indicated antibodies and protein G sepharose (GE Healthcare) or anti-FLAG M2 agarose beads (Sigma, A2220). For IP-qRT-PCR experiments, cells were lysed with PBS containing 0.1% Tween 20, 1% triton-X, 1-mM EDTA, protease inhibitor cocktail (Roche), and 2% glycerol. After centrifugation, crude lysates were subjected to anti-FLAG M2 beads for 4 h. Immune complexes were washed in lysis buffer 10 times and were then washed in lysis buffer containing an additional 100-mM NaCl. FLAG-Exosc3 and RNA complexes were eluted using free 3 × FLAG peptides (Sigma, F4799). Western blotting was performed using standard methods with rabbit anti-GAPDH (Sigma, G9545), mouse anti-FLAG (Sigma, F3165), rabbit anti-GFP (Clontech, 632376), anti-rabbit Igs HRP (Biosource, ALI3404), anti-rat Igs HRP (Jackson ImmunoResearch, 712-035-153), rabbit and mouse IgG TrueBlot (eBioscience, 18–8816, 18–8877), rat monoclonal anti-AID (MAID2, eBioscience, 14–5959), rabbit anti-A3G[38], anti-core (Dako, B0586), anti-human Exosc3 (GenWay Biotech, GNB-FF795C, F8130F), and isotype control (eBioscience 14–4321) antibodies. To generate a polyclonal antibody against AID, the C-terminal AID peptide (EVDDLRDAFRMLGF) was conjugated with cysteine and rabbits were immunized using keyhole limpet hemocyanin (KLH). Subsequently, anti-AID rabbit serum and rat monoclonal anti-AID were isolated and used in IP experiments. IgA class switching was determined by detecting surface IgA expression using flow cytometry as previously described[7,24,40].

### Quantitative PCR and RT-PCR

Total RNA was extracted using TRIsure (Bioline), was treated with DNase I (Takara) to eliminate transfected plasmids, and was then re-purified using TRIsure. For qRT-PCR analyses, 1 μg of total RNA was treated with amplification grade DNase I (Invitrogen) and was then reverse-transcribed using oligo-dT or random primers with SuperScript III (Invitrogen). Subsequently, cDNA was amplified using SYBR green ROX (Toyobo) with MX3000 (Stratagene) according to the PCR protocol. A1, AID, A3A, A3B, A3C, A3D, A3F, A3G, A3H, Exosc3, Exosc6, 18S ribosomal RNA, HPRT, and β-actin expression and HBV transcription were determined using PCR conditions of 95°C for 1 min followed by 40 cycles of 95°C for 15 s, 55°C for 30 s, and 70°C for 30 s, and one cycle of 95°C for 1 min, 55°C for 30 s, and 95°C for 30 s. For A3A amplification, an annealing temperature of 60°C was used. Copy numbers of APOBECs were determined using plasmid standard curves for each APOBEC (Fig 2A). Fold induction of APOBEC expression following treatment of cells with TGF-β1 was determined using the ΔΔCT method [43]. To eliminate transfected plasmids, purified NC-DNA from secreted virions and cytoplasmic lysates was obtained after serial DNase I digestion, proteinase K and SDS digestion, phenol–chloroform extraction, and isopropanol precipitation. NC-DNA copy numbers were determined using a HBV plasmid standard curve. Transcript expression levels in this study (except Fig 2A) are presented as fold induction relative to unstimulated cells. In transfection experiments, expression levels of mock-, GFP-, siGFP-, and siLuc-transfected cells were defined as one. Expression levels in qRT-PCR analyses were normalized to the amplification of internal controls (HPRT, β-actin, or 18S ribosomal RNA). Primers are listed in S2 Table.

### Statistical analysis

Differences were identified using the two-tailed unpaired Student’s *t*-tests and were considered significant when *P* < 0.05.

## Supporting Information

S1 Fig AID suppresses all HBV transcripts. (A) Schematic diagram of putative HBV transcripts. Structure of the HBV replicon plasmid (pPB) is shown on the top. Red arrows indicate the position of the X gene primers. The putative HBV transcripts are depicted on the bottom. (B, C) qRT-PCR analysis of HBV RNA. The RNA samples used in Fig 2B and 2E (lanes 4 and 8) were subjected to qRT-PCR analysis using the X gene primers. **P < 0.01 (*t*-test). Data are representative of two to three independent experiments and error bars represent standard errors of the mean.
(TIFF)

S2 Fig AID does not downregulate host cell gene expression. qRT-PCR analysis of cellular gene expression. The RNA samples used in Fig 2B were subjected to qRT-PCR analysis using the indicated gene primers. Expression levels of control GFP-expressing cells are defined as 1-fold. Error bars represent standard errors of the mean.
(TIFF)

S3 Fig AID binds to P protein in both the cytoplasm and nucleus in HBV replicating hepatocytes. Huh7 cells were transfected with the indicated expression vectors and pPB. Two days after transfection, cells were harvested and biochemically separated into three fractions (cytoplasmic, soluble nuclear, and insoluble nuclear fractions) using the Subcellular Protein Fractionation Kit (Thermo Scientific) as recommended by the manufacturer. Immunoprecipitation with FLAG agarose M2 beads was performed. Expected positions for AID-GFP and GFP proteins are indicated at the left side of the anti-GFP blot. PCNA is a putative soluble nuclear protein and was used as a control. Interaction of AID with P protein was determined by Western blot analysis.
(TIFF)

S4 Fig AID binds to Exosc3 in both the cytoplasm and nucleus in HBV replicating hepatocytes. Huh7 cells were transfected with the indicated expression vectors and pPB. Two days after transfection, cells were harvested and biochemically separated into three fractions (cytoplasmic, soluble nuclear, and insoluble nuclear fractions) using the Subcellular Protein Fractionation Kit (Thermo Scientific) as recommended by the manufacturer. Immunoprecipitation with FLAG agarose M2 beads was performed. Expected positions for AID-GFP and GFP proteins are indicated at the left side of the anti-GFP blot. Interaction of AID with Exosc3 was determined by Western blot analysis (A). Transcripts in the indicated fractions in A were subjected to RT-PCR analysis to determine coprecipitation of HBV and HPRT transcripts (B).
(TIFF)

S5 Fig Subcellular localization of the RNA exosome proteins. GFP expression of Huh7 transfectants used in Fig 5D was observed by fluorescence microscopy. GFP expression of the transfectants in Fig 5D (lanes 1–4 and 6–9) are shown in (A) and (B), respectively. GFP-tagged Exosc3, 2, and 7 are localized in both the cytoplasm and nucleus. A nuclear pattern of a GFP fusion protein is observed in some cells, especially in GFP-Exosc2- and-Exosc7-expressing cells.
(TIFF)

S6 Fig AID downregulates HBV RNA in the nucleus. Huh7 cells were transfected with an AID-GFP (or GFP) expression vector and pPB. Two days after transfection, cells were harvested and biochemically separated into three fractions (soluble cytoplasmic, soluble nuclear, and whole cell extract) using the Subcellular Protein Fractionation Kit (Thermo Scientific) as recommended by the manufacturer. Expression of AID-GFP, GFP, and PCNA were detected by western blot (A) and HBV RNA levels were determined by qRT-PCR analysis (B). *P < 0.05, **P < 0.01 (*t*-test), error bars represent standard errors of the mean. Levels of HBV RNA from GFP transfectants were defined as one.
(TIFF)

S7 Fig cccDNA level in Huh7 cells transfected with HBV replicon plasmids. Rolling circle amplification (RCA) is capable of amplifying circular DNA such as HBV plasmid and cccDNA. cccDNA production in pPB-transfected Huh7 cells and 7T7-8 cells was compared with that in cccDNA-producing cells (HepG2.2.15). (**A**) Schematic diagram of RCA and analysis of cccDNA is shown. (**B**) Huh7 cells were transfected with pPB (or pPB-dP) and cultivated for 3 days. Huh7T7-8 cells were cultivated in the absence of tetracycline for 3 days. HepG2.2.15 cells were used as a cccDNA producing control cells. Nuclear fraction of each transfectant was subjected to Hirt extraction to extract cccDNA. cccDNA was amplified by RCA. As a standard reaction, 10^7^, 10^8^, and 10^9^ copies of HBV plasmids were amplified side by side as a standard reaction. Amplified RCA products were digested by EcoRI (for plasmid standard reactions, EcoRV) and agarose electrophoresis image visualized by ethidium bromide is shown (top). EcoRI digestion converts concatemeric cccDNA into 3.2 kb monomer, while EcoRV digestion converts concatemeric HBV plasmids into 3.2-kb and 4.2-kb DNA. Nucleocapsid production of each transfectants was also determined by extraction of nucleocapsid RC-DNA following PCR detection of HBV DNA (bottom).
(TIFF)

S8 Fig Knocking down of ZAP expression did not affect AID-mediated downregulation of HBV transcripts. Huh7 cells were transfected with an AID (or GFP) expression vector with indicated siRNA together with pPB. Cells were harvested after 3 days of incubation. Levels of ZAP expression and HBV RNA were determined by RT-qPCR. siZAP-1 and -2 were obtained from Invitrogen. siZAP-3 [37] was obtained from Santa Cruz Biotechnology. (A) Fluorescence microscopic image of GFP transfectants on day 3 after transfection. Knocking down of GFP expression is obvious in siGFP transfectants. (B) ZAP mRNA expression levels. The expression level of ZAP in the GFP-transfected, GFP siRNA (siGFP)-transfected (far right) was defined as one. (C) HBV RNA levels: HBV RNA level in the GFP-transfected, GFP siRNA (siGFP)-treated (far right) was defined as one. AID expression reduces HBV RNA levels in both siZAP and siGFP transfectants. (D) HBV RNA levels: The same data set in (C) was plotted with HBV RNA levels in siGFP-AID and siGFP-GFP transfectants defined as one. Knocking down of ZAP increases HBV RNA levels in both AID and GFP transfectants. **P < 0.01 (*t*-test), error bars represent standard errors of the mean.
(TIFF)

S1 Table List of plasmids used in this study. (PDF)

S2 Table List of primers used in this study. (PDF)

S3 Table List of Genbank accession numbers used in this study. (PDF)
