# Supplementary figures and images for: TGF-β Suppression of HBV RNA through AID-Dependent Recruitment of an RNA Exosome Complex
Source: PLoS Pathog. 2015 Apr 2;11(4):e1004780. doi: 10.1371/journal.ppat.1004780 (PMC4383551; doi:10.1371/journal.ppat.1004780)

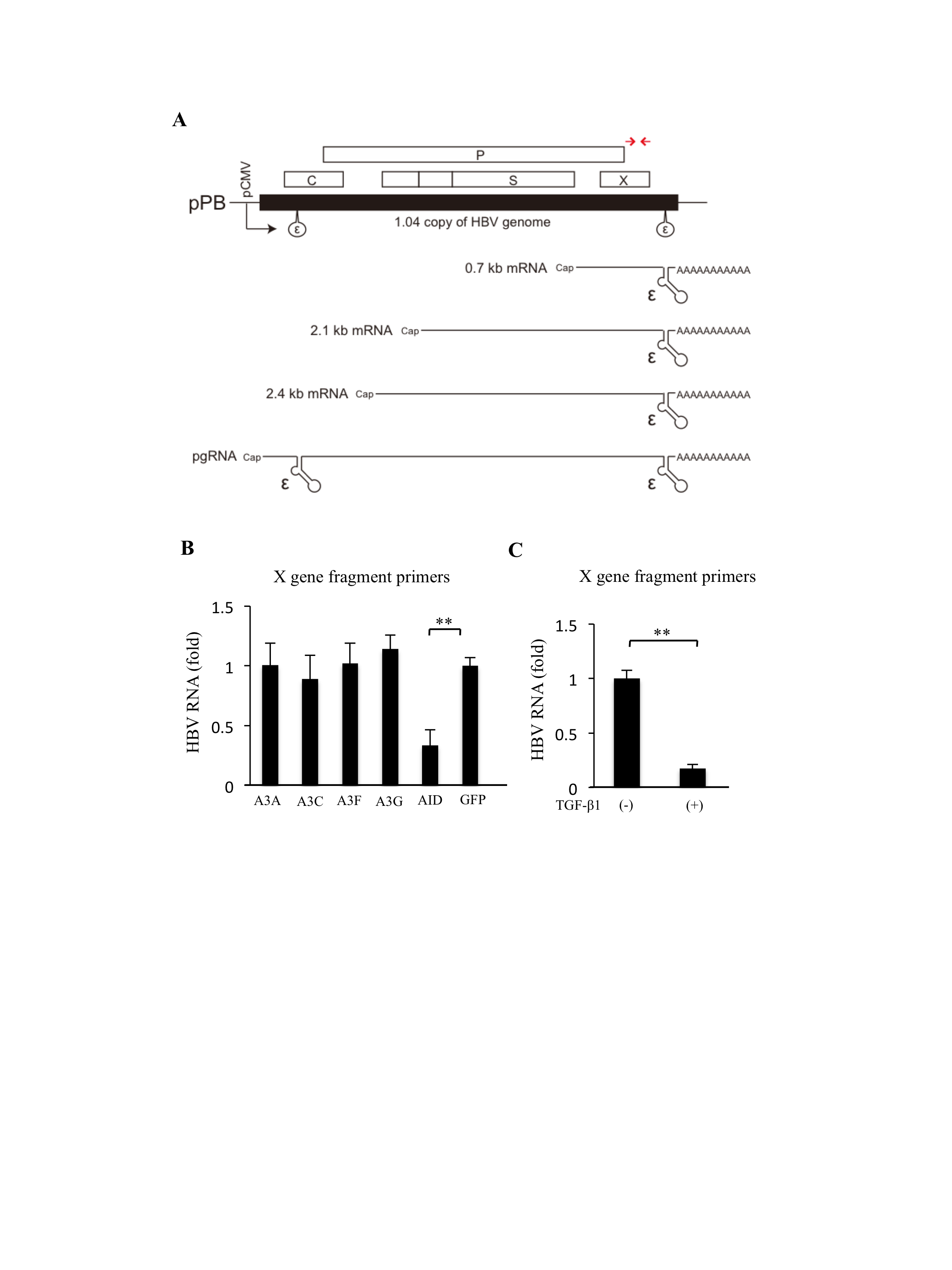

Supplement: S1 Fig — (A) Schematic diagram of putative HBV transcripts. Structure of the HBV replicon plasmid (pPB) is shown on the top. Red arrows indicate the position of the X gene primers. The putative HBV transcripts are depicted on the bottom. (B, C) qRT-PCR analysis of HBV RNA. The RNA samples used in Fig 2B and 2E (lanes 4 and 8) were subjected to qRT-PCR analysis using the X gene primers. **P < 0.01 (t-test). Data are representative of two to three independent experiments and error bars represent standard errors of the mean. (TIFF) [file ppat.1004780.s001.tiff]

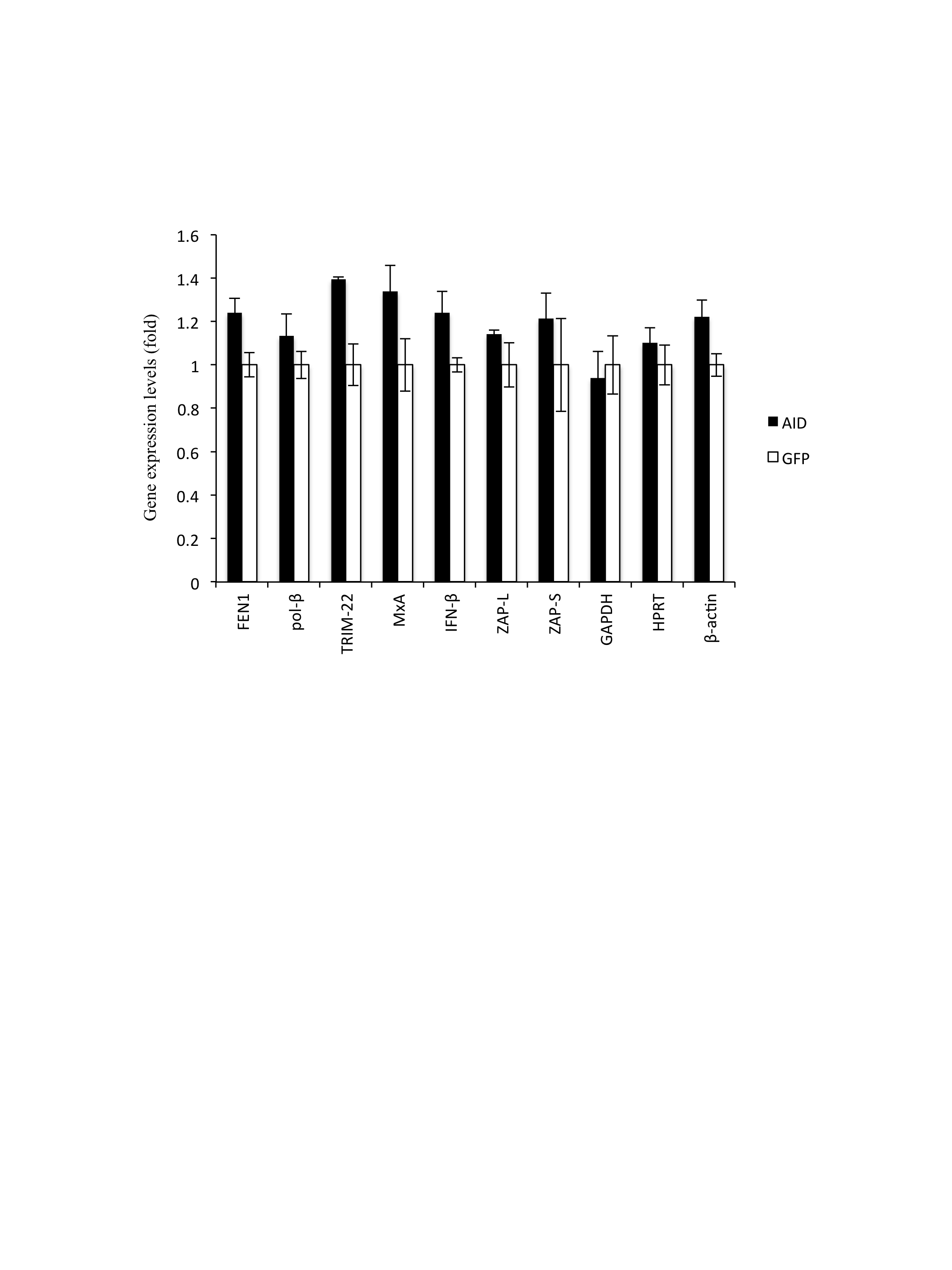

Supplement: S2 Fig — qRT-PCR analysis of cellular gene expression. The RNA samples used in Fig 2B were subjected to qRT-PCR analysis using the indicated gene primers. Expression levels of control GFP-expressing cells are defined as 1-fold. Error bars represent standard errors of the mean. (TIFF) [file ppat.1004780.s002.tiff]

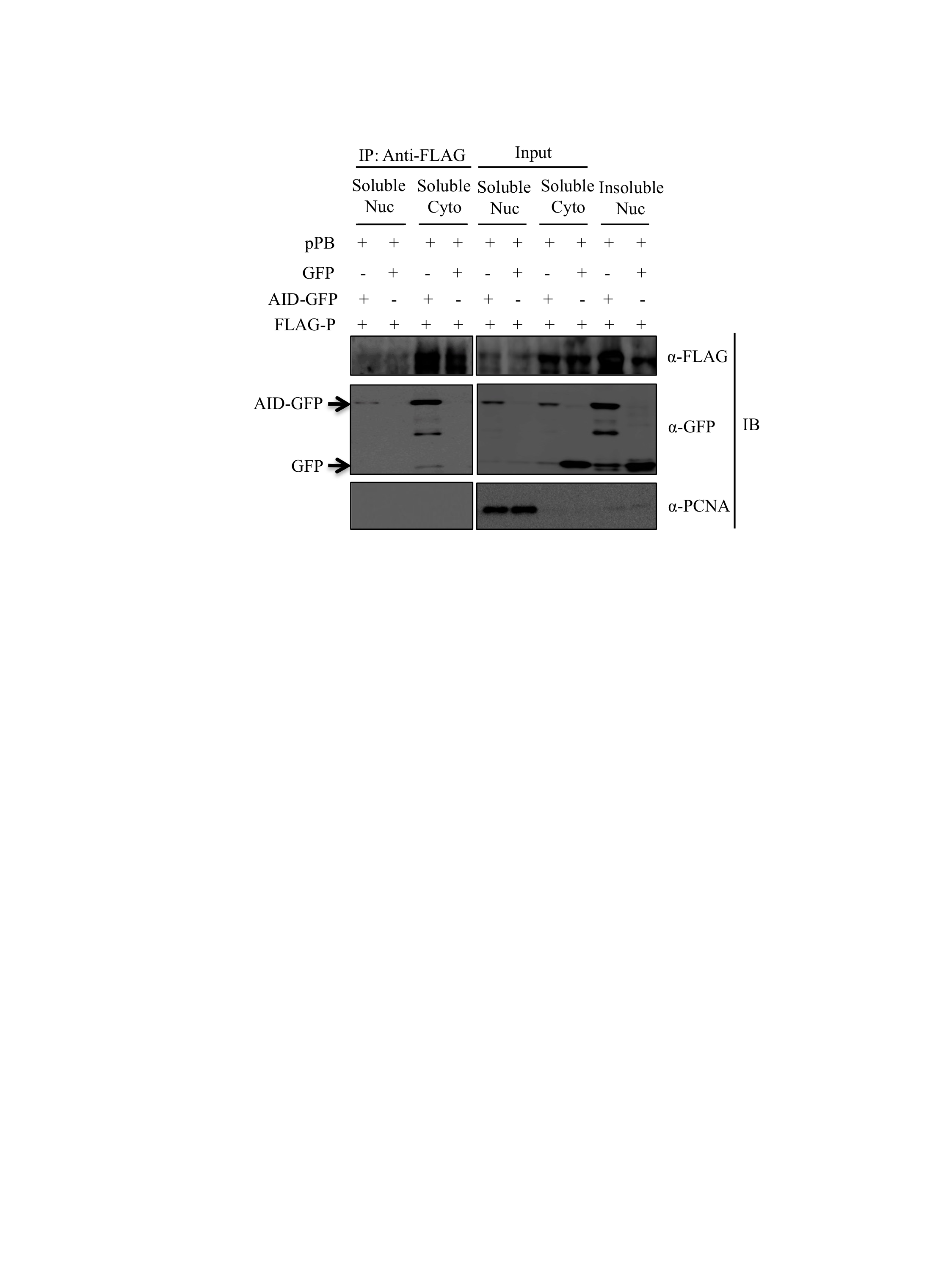

Supplement: S3 Fig — Huh7 cells were transfected with the indicated expression vectors and pPB. Two days after transfection, cells were harvested and biochemically separated into three fractions (cytoplasmic, soluble nuclear, and insoluble nuclear fractions) using the Subcellular Protein Fractionation Kit (Thermo Scientific) as recommended by the manufacturer. Immunoprecipitation with FLAG agarose M2 beads was performed. Expected positions for AID-GFP and GFP proteins are indicated at the left side of the anti-GFP blot. PCNA is a putative soluble nuclear protein and was used as a control. Interaction of AID with P protein was determined by Western blot analysis. (TIFF) [file ppat.1004780.s003.tiff]

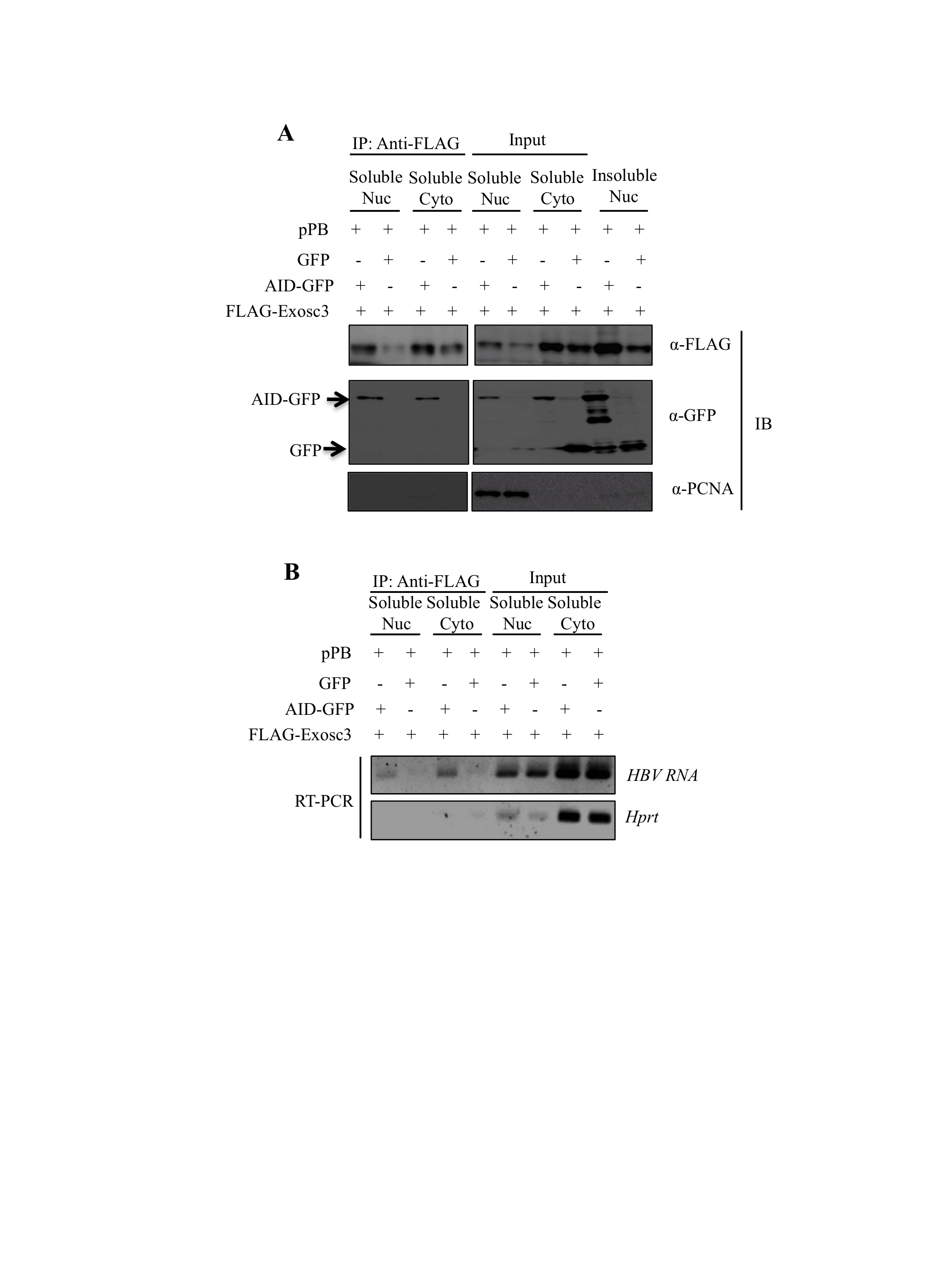

Supplement: S4 Fig — Huh7 cells were transfected with the indicated expression vectors and pPB. Two days after transfection, cells were harvested and biochemically separated into three fractions (cytoplasmic, soluble nuclear, and insoluble nuclear fractions) using the Subcellular Protein Fractionation Kit (Thermo Scientific) as recommended by the manufacturer. Immunoprecipitation with FLAG agarose M2 beads was performed. Expected positions for AID-GFP and GFP proteins are indicated at the left side of the anti-GFP blot. Interaction of AID with Exosc3 was determined by Western blot analysis (A). Transcripts in the indicated fractions in A were subjected to RT-PCR analysis to determine coprecipitation of HBV and HPRT transcripts (B). (TIFF) [file ppat.1004780.s004.tiff]

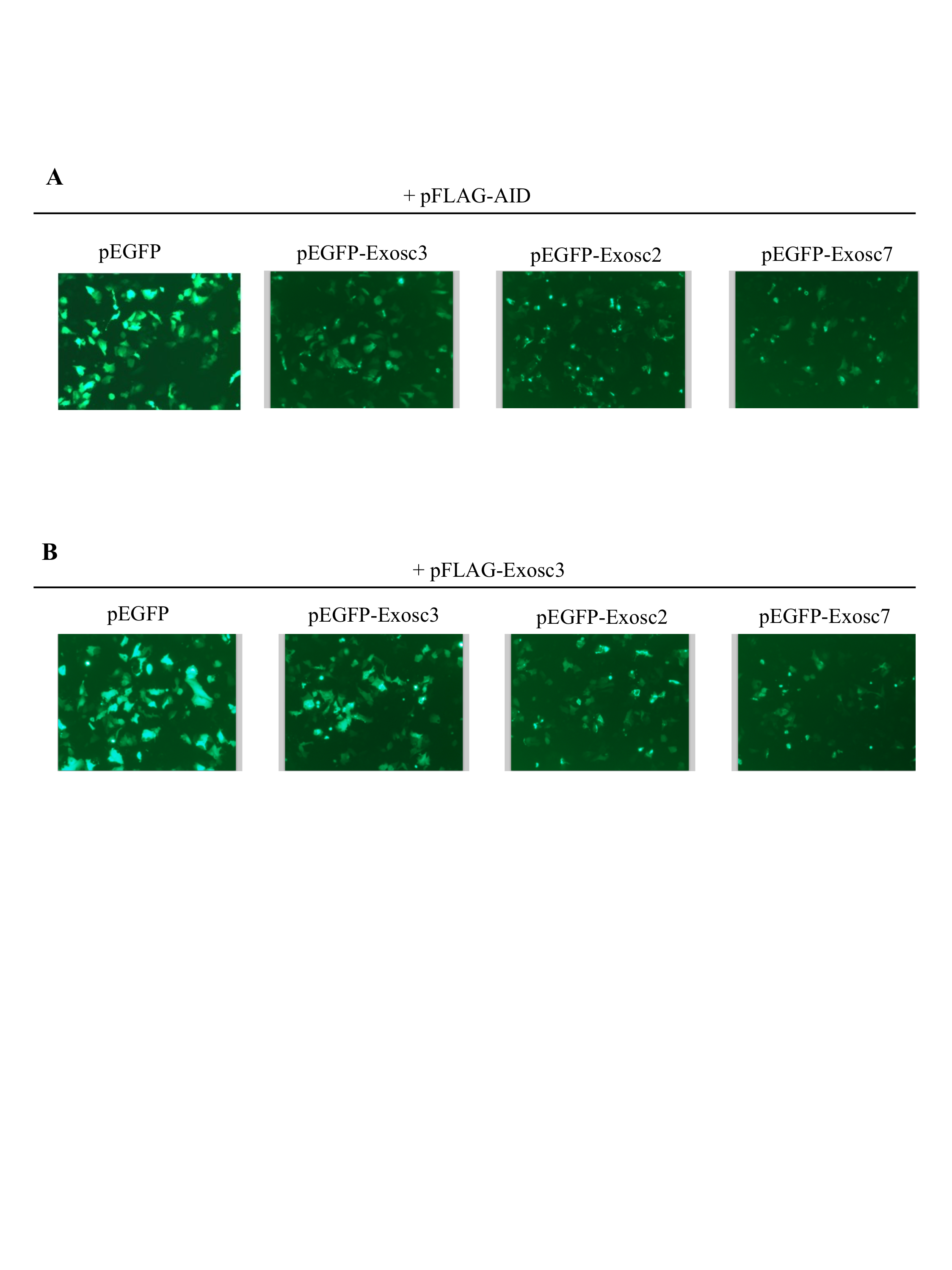

Supplement: S5 Fig — GFP expression of Huh7 transfectants used in Fig 5D was observed by fluorescence microscopy. GFP expression of the transfectants in Fig 5D (lanes 1–4 and 6–9) are shown in (A) and (B), respectively. GFP-tagged Exosc3, 2, and 7 are localized in both the cytoplasm and nucleus. A nuclear pattern of a GFP fusion protein is observed in some cells, especially in GFP-Exosc2- and-Exosc7-expressing cells. (TIFF) [file ppat.1004780.s005.tiff]

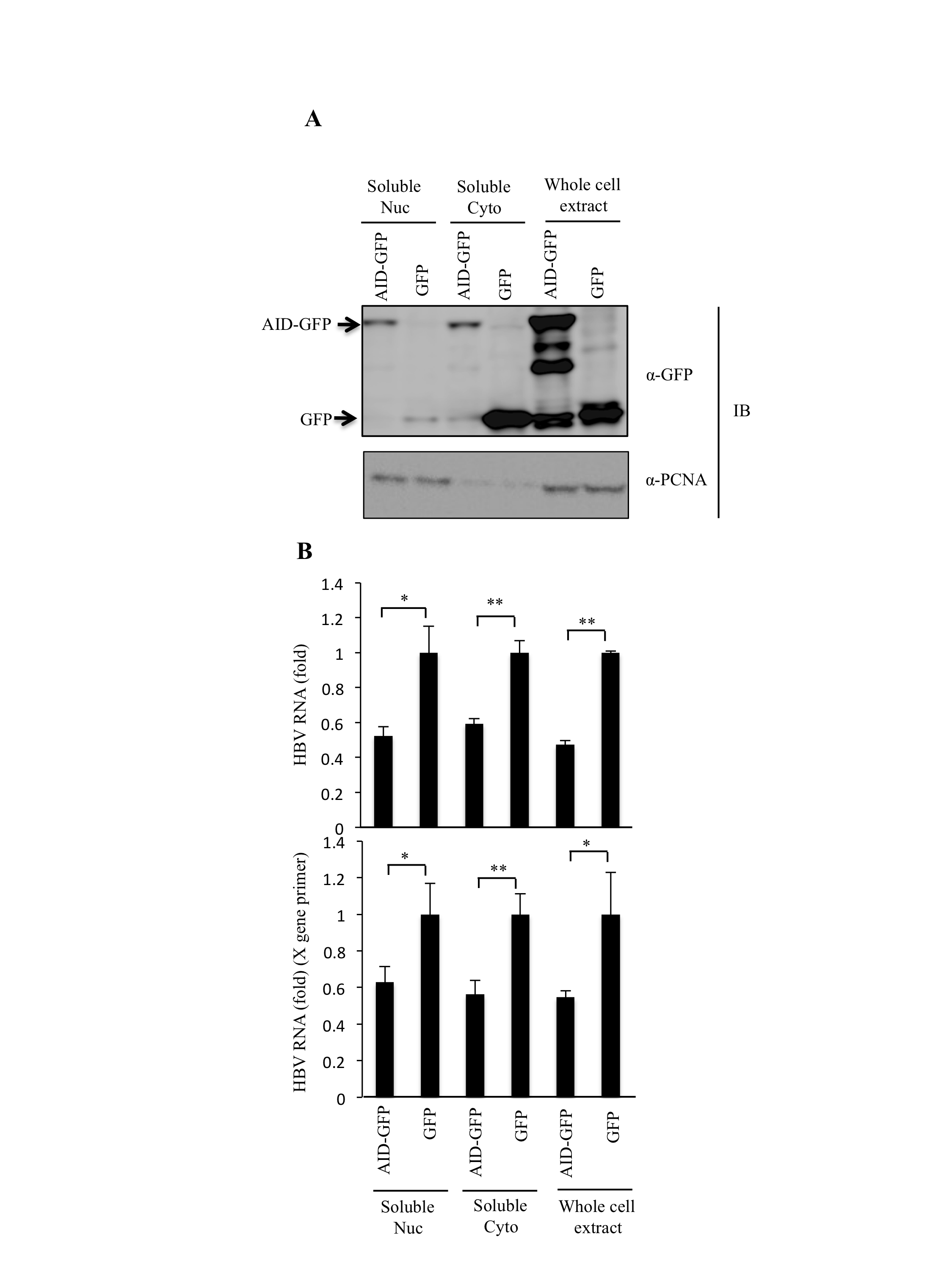

Supplement: S6 Fig — Huh7 cells were transfected with an AID-GFP (or GFP) expression vector and pPB. Two days after transfection, cells were harvested and biochemically separated into three fractions (soluble cytoplasmic, soluble nuclear, and whole cell extract) using the Subcellular Protein Fractionation Kit (Thermo Scientific) as recommended by the manufacturer. Expression of AID-GFP, GFP, and PCNA were detected by western blot (A) and HBV RNA levels were determined by qRT-PCR analysis (B). *P < 0.05, **P < 0.01 (t-test), error bars represent standard errors of the mean. Levels of HBV RNA from GFP transfectants were defined as one. (TIFF) [file ppat.1004780.s006.tiff]

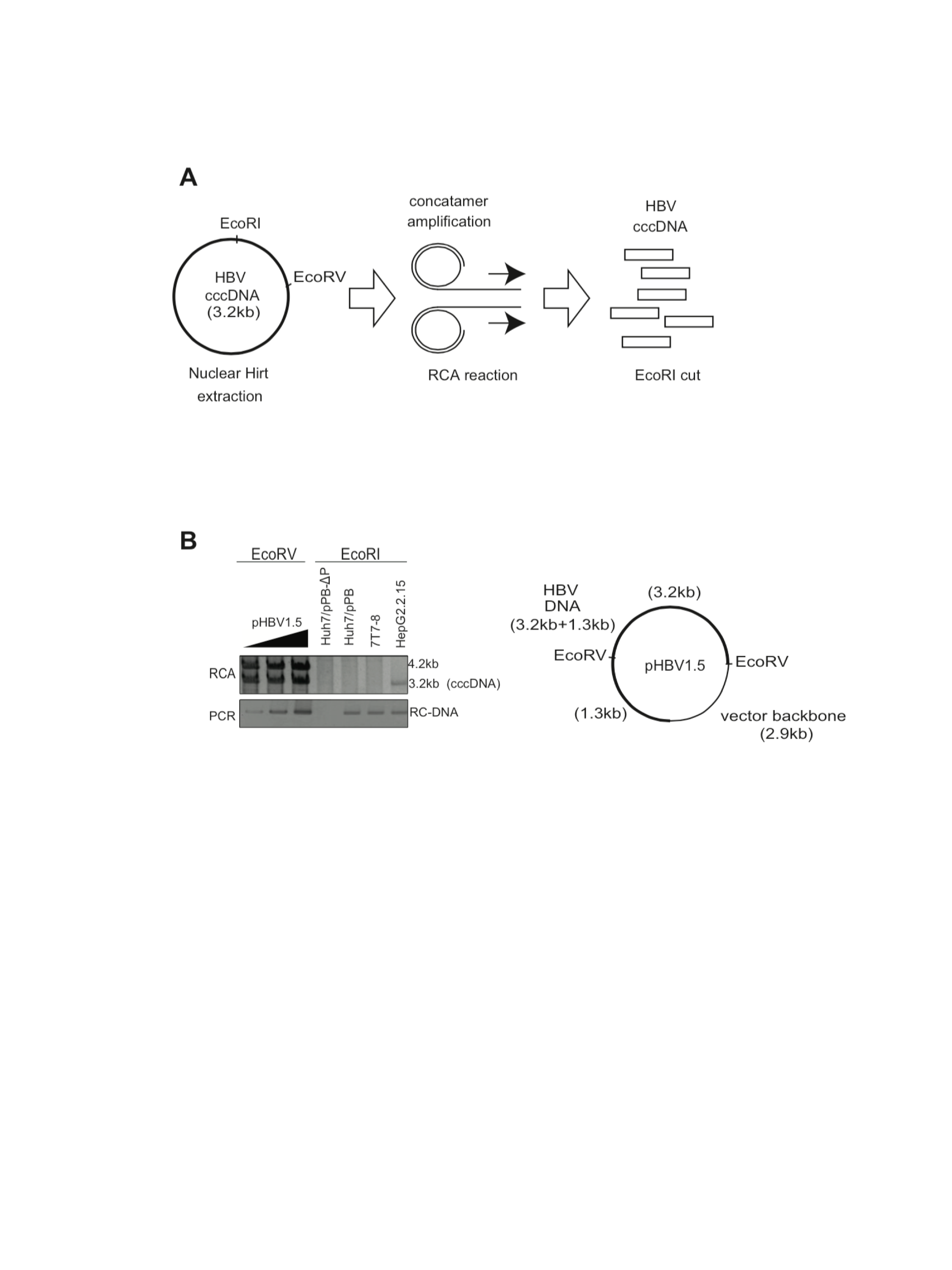

Supplement: S7 Fig — Rolling circle amplification (RCA) is capable of amplifying circular DNA such as HBV plasmid and cccDNA. cccDNA production in pPB-transfected Huh7 cells and 7T7-8 cells was compared with that in cccDNA-producing cells (HepG2.2.15). (A) Schematic diagram of RCA and analysis of cccDNA is shown. (B) Huh7 cells were transfected with pPB (or pPB-dP) and cultivated for 3 days. Huh7T7-8 cells were cultivated in the absence of tetracycline for 3 days. HepG2.2.15 cells were used as a cccDNA producing control cells. Nuclear fraction of each transfectant was subjected to Hirt extraction to extract cccDNA. cccDNA was amplified by RCA. As a standard reaction, 107, 108, and 109 copies of HBV plasmids were amplified side by side as a standard reaction. Amplified RCA products were digested by EcoRI (for plasmid standard reactions, EcoRV) and agarose electrophoresis image visualized by ethidium bromide is shown (top). EcoRI digestion converts concatemeric cccDNA into 3.2 kb monomer, while EcoRV digestion converts concatemeric HBV plasmids into 3.2-kb and 4.2-kb DNA. Nucleocapsid production of each transfectants was also determined by extraction of nucleocapsid RC-DNA following PCR detection of HBV DNA (bottom). (TIFF) [file ppat.1004780.s007.tiff]

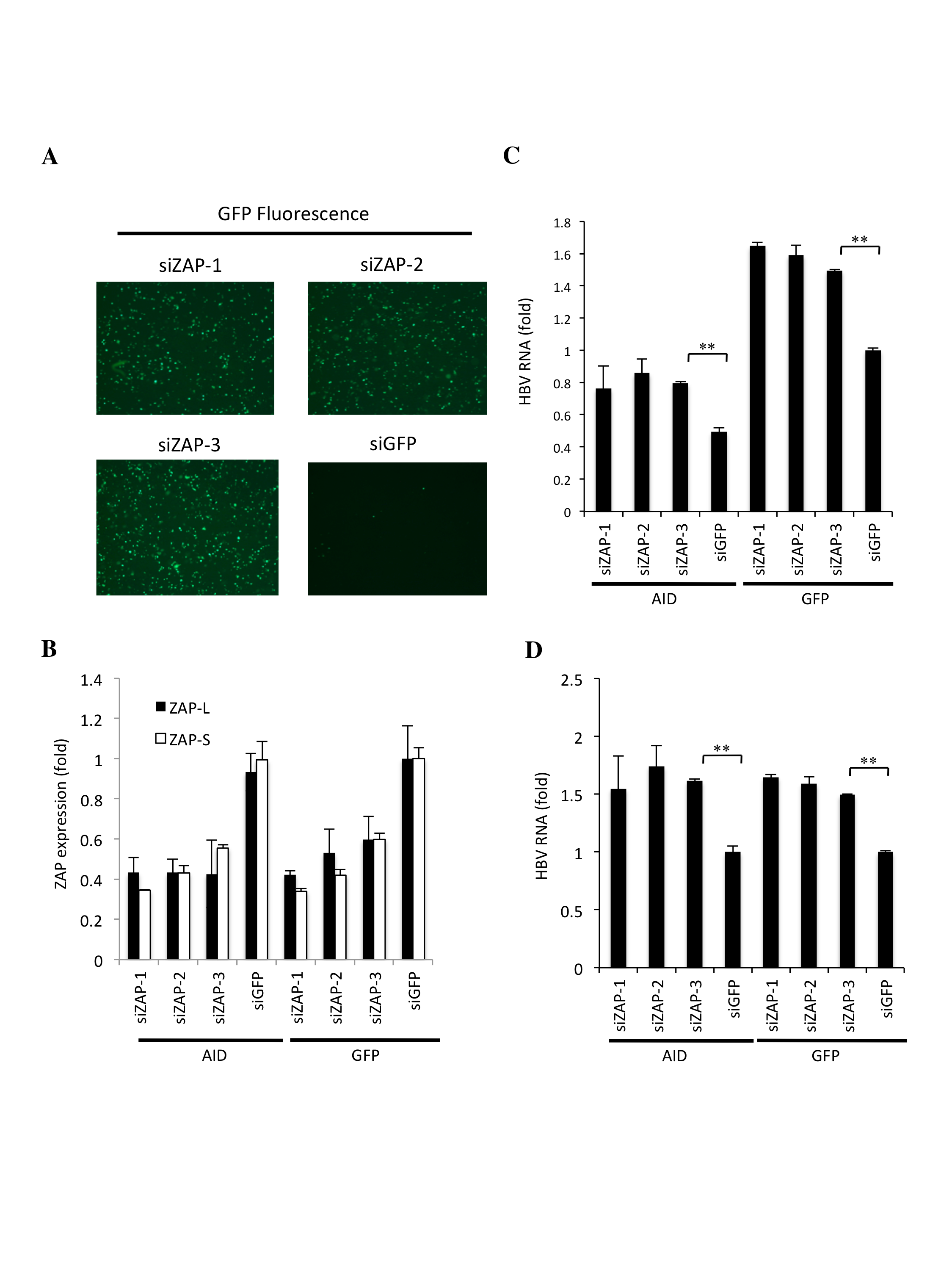

Supplement: S8 Fig — Huh7 cells were transfected with an AID (or GFP) expression vector with indicated siRNA together with pPB. Cells were harvested after 3 days of incubation. Levels of ZAP expression and HBV RNA were determined by RT-qPCR. siZAP-1 and -2 were obtained from Invitrogen. siZAP-3 [37] was obtained from Santa Cruz Biotechnology. (A) Fluorescence microscopic image of GFP transfectants on day 3 after transfection. Knocking down of GFP expression is obvious in siGFP transfectants. (B) ZAP mRNA expression levels. The expression level of ZAP in the GFP-transfected, GFP siRNA (siGFP)-transfected (far right) was defined as one. (C) HBV RNA levels: HBV RNA level in the GFP-transfected, GFP siRNA (siGFP)-treated (far right) was defined as one. AID expression reduces HBV RNA levels in both siZAP and siGFP transfectants. (D) HBV RNA levels: The same data set in (C) was plotted with HBV RNA levels in siGFP-AID and siGFP-GFP transfectants defined as one. Knocking down of ZAP increases HBV RNA levels in both AID and GFP transfectants. **P < 0.01 (t-test), error bars represent standard errors of the mean. (TIFF) [file ppat.1004780.s008.tiff]
