# Supplementary material for: TGF-β Suppression of HBV RNA through AID-Dependent Recruitment of an RNA Exosome Complex
Source: PLoS Pathog. 2015 Apr 2;11(4):e1004780. doi: 10.1371/journal.ppat.1004780 (PMC4383551; doi:10.1371/journal.ppat.1004780)
Supplement: S1 Table — (PDF) [file ppat.1004780.s009.pdf]

pFLAGhA3A	cDNA encoding human A3A (BC126416) was purchased from Open Biosystems. A3A ORF was generated by PCR with 5'-AATTCATGGAAGCCAGCCCAGC-3' and 5'-ATATCTCGAGTCAGTTCCCTGATTCT-3' and inserted to an EcoRI/SalI site in pcDNA3Tage1A (Invitrogen).
pFLAGhA3C	An open reading frame of A3C(NM_014508) was produced by RT-PCR with 5'-AAGAATGAATCCACAGATCAGA-3' and 5'-AAAACTCGAGTCACTGGAGACTCTCCCGT-3' primers, using cDNA derived from Huh7 cells. The fragment was digested by EcoRI and XhoI and cloned in pEGFP-C2 (Clonetech).
pFLAGhA3G	previously mentioned (36)
pFLAGhA3F	An open reading frame of A3F (NM_145298) was produced by RT-PCR with 5'-ATTTAAGTGAAGCCTCACTTCAG-3' and 5'-AATTCTCGAGTCATTCGAGAATCTCCTGCAG-3' primers, using cDNA derived from Huh7 cells. The fragment was digested by EcoRI and XhoI and cloned in pGFP2.
p3flag-GFP	previously mentioned [(26) (36)]
pCMV-hAIDGFP	previously mentioned [(26)]
pCMV-p19GFP	p19-AID (39) open reading frame was inserted into pEGFP -N1
pPB	previously mentioned [(26) (36)]
pTre-HBV	A replication-competent HBV reolicon plasmid, in which the HBV pgRNA is driven by tet promoter previously mentioned (Tsuge et al.,2005)
pTETtTAK zeo	previously mentioned (26)
pPB-ΔP	previously mentioned (26)
pFLAG-P	previously mentioned (26)
pFLAG-PΔC	DNA region between Van91 and SpeI sites which encodes the C-terminal portion of P protein was deleted from pFLAG-P.
pFLAG-AID	Human AID open reading frame was inserted in a multiple cloning site of pCMV3TAG3B.
pEGFP-Exosc3	An open reading frame of human Exosc3 (NM_016042) was produced by RT-PCR with 5'-AAAGAATTCATGGCCGAACCTGCGTCTGTC-3' and 5'-TTTCTCGAGCTATATCAACTTTCTGCCAAT-3' primers, using cDNA derived from Huh7 cells. The fragment was digested by EcoRI and XhoI and cloned in pEGFP-C2.
pEGFP-Exosc2	An open reading frame of human Exosc2 (NM_014285) was produced by RT-PCR with 5'-GAATTCATGGAGATGAGGCTTCCAGTG-3' and 5'-CTCGAGTTATCCCTCCTGTTCCAAAA-3' primers, using cDNA derived from Huh7 cells. The fragment was digested by EcoRI and XhoI and cloned in pEGFP-C2.
pEGFP-Exosc7	An open reading frame of human Exosc7 (XR_245104) was produced by RT-PCR with 5'-GAATTCATGGCGTCCGTGACGCTGAG-3' and 5'-CTCGAGTCATCCCAGGAATCCAACTT-3' primers, using cDNA derived from Huh7 cells. The fragment was digested by EcoRI and XhoI and cloned in pEGFP-C2.
pCMV-hAID	previously mentioned (26)
pFLAG-Exosc3	An open reading frame of Exosc3 (NM_016042) was produced by RT-PCR with 5'-AAAGAATTCATGGCCGAACCTGCGTCTGTC-3' and 5'-TTTCTCGAGCTATATCAACTTTCTGCCAAT-3' primers, using cDNA derived from Huh7 cells. The fragment was digested by EcoRI and XhoI and cloned in pCMV-3Tag1A.
shAID-pLKO1puro	perchased from Thermo Scientific. (TRCN0000050345)
shExosc3-pLKO1puro	perchased from Thermo Scientific. (TRCN0000050410)
shLuc-pLKO1puro	5'-CCGGCGCTGAGTACTTCGAAATGTCCTCGAGGACATTTCGAAGTACTCAGCGTTTTTG-3' and 5'-AATTCAAAAACGCTGAGTACTTCGAAATGTCCTCGAGGACATTTCGAAGTACTCAGCG-3', were hybridized and inserted in an Agel/EcoRl site of pLKO1puro (Thermo Scientific)
pHBV1.5	previously mentioned[(26) (36)]
pGFP-P	A P gene open reading frame in p3flag-P is subcloned into pEGFP-C2 in frame by XhoI and HindIII, so that transfection of this plasmid causes to express GFP-tagged P protein.
pDsRed	previously mentioned[(34)]
	Successful construction of the vector was verified by DNA sequencing.
