# Supplementary material for: TGF-β Suppression of HBV RNA through AID-Dependent Recruitment of an RNA Exosome Complex
Source: PLoS Pathog. 2015 Apr 2;11(4):e1004780. doi: 10.1371/journal.ppat.1004780 (PMC4383551; doi:10.1371/journal.ppat.1004780)
Supplement: S2 Table — (PDF) [file ppat.1004780.s010.pdf]

Name	Primer sequence
HBV for1	GAATTGATGACTCTAGCTACCTG
HBV rev1	GAAACCACAATAGTTGCCTGATC
A3A for1	ATGGCATTGGAAGGCATAAG
A3A rev1	CAAAGAAGGAACCAGGTCCA
A3B for1	TTCGAGGCCAGGTGTATTTCA
A3B rev1	CAGAGATGGTCAGGGTGACA
A3C for1	CAACGATCGGAACGAAACTT
A3C rev1	TATGTCGTCGCAGAACCAAG
A3D for1	ACCCAAACGTCAGTCGAATC
A3D rev1	GCTCAGCCAAGAATTTGGTC
A3F for1	GAAACACAGTGGAGCGAATG
A3F rev1	GAAATGGGGCTCTGATGAAAG
A3G for1	GGTCAGAGGACGGCATGAGA
A3G rev1	GCAGGACCCAGGTGTCATTG
A3H for1	CCCGCCTGTACTACCACTGG
A3H rev1	GGGTTGAAGGAAAGCGGTTT
hAID for1	AAATGTCCGCTGGGCTAAGG
hAID rev1	GGAGGAAGAGCAATTCCACGT
HPRT for1	GCCCTGGCGTCGTGATTAGT
HPRT rev1	CGAGCAAGACGTTCAGTCCTGTC
18s for1	TACCTGGTTGATCCTGCCAGTAGC
18s rev1	AACTGATTTAATGAGCCATTCGC
Exosc3 for1	GGAGATCTCATCTATGGCCA
Exosc3 rev1	CATGTGTTCACAAGCTTCT
Exosc6 for1	GAACCAGAAGCCTGAGCAAC
Exosc6 rev1	CAGACCAAGTCCCACCATCT
5sRNA for1	TACGGCCATACCACCCTGAA
5sRNA rev1	GCGGTCTCCCATCCAAGTAC
mAID for1	GGAACAGCATAACTTCCAGACTTTG
mAID rev1	CCTGAAAGTGAGCCTTAGAGGGAA
m-b-actin for1	CTGGAGAAGAGCTATGAGCTGC
m-b-actin rev1	CAACGTCACACTTCATGATGG
mGAPDH for1	TGAAGCAGGCATCTGAGGG
mGAPDH rev1	CGAAGGTGGAAGAGTGGGAG
pTre-HBV for1	TCCAGATTGGGACTTCAACC
pTre-HBV rev1	CTGCTGGCACTGTTGTCAAT
GAPDH for1	TGCACCACCAACTGCTTAGC
GAPDH rev1	GGCATGGACTGTGGTCATGAG
HBV for2	CGGAAATATACATCGTTTCCAT
HBV rev2	AAGAGTCCTCTTATGTAAGACCTT
HBV X for	ATGGCTGCTAGGCTGTACTGCCAA
HBV X rev	TGAGAAGGCACAGACGGGGAGA
FEN1 for	CTGTGGACCTCATCCAGAAGCA
FEN1 rev	CCAGCACCTCAGGTTCCAAGA
pol-β for	ACGTAAACTGGAAAAGATTCGGC

pol-β rev	GCCCAATTCGCTGATGATGGTTC
TRIM-22 for	GGTTGAGGGGATCGTCAGTA
TRIM-22 rev	TTGGAAACAGATTTTGGCTTC
MxA for	AAGCTGATCCGCCTCCACTT
MxA rev	TGCAATGCACCCCTGTATACC
IFN-β for	GGCTGGCCCTGTGATATTTCTGTG
IFN-β rev	ACCTGGCTCTCCTCCTCCCTTCCT
ZAP-L for	GCTGAGTTTCCAAGGGATGAT
ZAP-L rev	AGTCCTCCTGAGGACGAAAGG
ZAP-S for	GCTGAGTTTCCAAGGGATGAT
ZAP-S rev	GGCTCCAGATTCACGAGTGAC
