# Supplementary material for: TGF-β Suppression of HBV RNA through AID-Dependent Recruitment of an RNA Exosome Complex
Source: PLoS Pathog. 2015 Apr 2;11(4):e1004780. doi: 10.1371/journal.ppat.1004780 (PMC4383551; doi:10.1371/journal.ppat.1004780)
Supplement: S3 Table — (PDF) [file ppat.1004780.s011.pdf]

Gene	Accession Number
HBV	X02763
hAID	NM_020661.2
HBV P	X02763
A3A	NM_001270406.1
A3B	NM_004900.4
A3C	NM_014508.2
A3F	NM_145298.5
A3G	NM_021822.3
mAID	NM_009645.2
EXOSC2	NM_014285.6
EXOSC3	NM_016042.3
EXOSC6	NM_058219.2
EXOSC7	NM_015004.3
FEN1	NM_004111.5
HPRT	M26434.1
GAPDH	NM_002046.5
Pol-β	NM_002690.2
TRIM-22	NM_006074.4
MxA	NM_001144925.2
IFN-β	NM_002176.2
β-actin	X00351.1
Zap-L	NP_064504.2
Zap-S	NP_078901.3
